# Gender Differences in Searching for Health Information on the Internet and the Virtual Patient-Physician Relationship in Germany: Exploratory Results on How Men and Women Differ and Why

**DOI:** 10.2196/jmir.4127

**Published:** 2015-06-22

**Authors:** Sonja Bidmon, Ralf Terlutter

**Affiliations:** ^1^ Department of Marketing and International Management Alpen-Adria-Universitaet Klagenfurt Klagenfurt am Woerthersee Austria

**Keywords:** gender differences, physician-patient relations, information seeking behavior, general practitioners, statistical factor analysis

## Abstract

**Background:**

Many studies have shown that women use the Internet more often for health-related information searches than men, but we have limited knowledge about the underlying reasons. We also do not know whether and how women and men differ in their current use of the Internet for communicating with their general practitioner (GP) and in their future intention to do so (virtual patient-physician relationship).

**Objective:**

This study investigates (1) gender differences in health-related information search behavior by exploring underlying emotional, motivational, attitudinal as well as cognitive variables, situational involvement, and normative influences, and different personal involvement regarding health-related information searching and (2) gender differences in the virtual patient-physician relationship.

**Methods:**

Gender differences were analyzed based on an empirical online survey of 1006 randomly selected German patients. The sample was drawn from an e-panel maintained by GfK HealthCare. A total of 958 usable questionnaires were analyzed. Principal component analyses were carried out for some variables. Differences between men (517/958) and women (441/958) were analyzed using t tests and Kendall’s tau-b tests. The survey instrument was guided by several research questions and was based on existing literature.

**Results:**

Women were more engaged in using the Internet for health-related information searching. Gender differences were found for the frequency of usage of various Internet channels for health-related information searches. Women used the Internet for health-related information searches to a higher degree for social motives and enjoyment and they judged the usability of the Internet medium and of the information gained by health information searches higher than men did. Women had a more positive attitude toward Web 2.0 than men did, but perceived themselves as less digitally competent. Women had a higher health and nutrition awareness and a greater reluctance to make use of medical support, as well as a higher personal disposition of being well-informed as a patient. Men may be more open toward the virtual patient-physician relationship.

**Conclusions:**

Women have a stronger social motive for and experience greater enjoyment in health-related information searches, explained by social role interpretations, suggesting these needs should be met when offering health-related information on the Internet. This may be interesting for governmental bodies as well as for the insurance and the pharmaceutical industries. Furthermore, women may be more easily convinced by health awareness campaigns and are, therefore, the primary target group for them. Men are more open to engaging in a virtual relationship with the GP; therefore, they could be the primary target group for additional online services offered by GPs. There were several areas for GPs to reinforce the virtual patient-physician relationship: the fixing of personal appointments, referral to other doctors, writing prescriptions, and discussions of normal test results and doctor’s notes/certificates of health.

## Introduction

### General Background

The Internet is one of the most important sources of health information, no longer only for a small segment of Internet users, but now also for the “general public” [1]. According to the Pew Internet & American Life Project [2], the most prominent opinion poll about health-related information searches on the Internet, gender is one of the most important predictors of seeking health information on the Internet [1,3]. Over a vast span of empirical studies (eg, [2-8]), it has been demonstrated that women are more likely than men to look for health information on the Internet. However, the research so far has focused on the frequency of health information searching rather than on the underlying constructs that may help to explain such differences in Internet health information searching. Thus, this paper goes beyond existing literature by analyzing possible reasons for gender differences in Internet health information search behavior. The paper addresses gender differences in motives, emotions, cognitions, situational, and personal involvement with regard to health-related information searching.

Furthermore, the second part of the paper deals with whether and how men and women differ with regard to the virtual patient-physician relationship. In our paper, we define the virtual patient-physician relationship as communication between a patient and the physician (or the physician’s surgery or office) via the Internet. Examples include emailing, making an appointment online to see the doctor, and a virtual meeting with the doctor (eg, via Skype). We address current communication as well as future intention to communicate with the general practitioner (GP) via the Internet in general and with regard to different areas of treatment (eg, routine treatments, acute disorders, discussion of health test results, referrals to other physicians).

### Theoretical Background and Research Questions

There are many approaches and models that aim at explaining why individuals search for information. For instance, Marton and Choo [1] analyzed 4 theoretically grounded quantitative studies of health information seeking on the Internet and found that the multidisciplinary frameworks differ substantially. In addition, information technology research has yielded many different competing models of users’ acceptance of new technologies (see for an overview Venkatesh et al [9]), each with a different focus and a different set of antecedents of technology adoption. With regard to the main focus of this paper to investigate gender differences in health-related information search behavior on the Internet as well as in the virtual patient-physician relationship, 3 models seem to be particularly appropriate as a theoretical basis: the theory of planned behavior (TPB) [10,11], the technology acceptance model (TAM) [12-14], and the functional theory of media use [15].

An extension of the theory of reasoned action (TRA) [16,17], TPB proposes a causal relationship of the exogenous variables attitude toward behavior, subjective norm, and perceived behavioral control with the endogenous variable behavioral intention [10,11]. Behavioral intention and perceived behavioral control together influence behavior. Based on the TPB, Venkatesh et al [18] found that there are clear gender differences in the salience of factors influencing the decision to adopt a new technology in the workplace. According to these authors, the “role of gender in technology adoption and usage behavior is crucial” [18]. These differences could be observed even when controlling several confounding variables, such as income, education, or digital literacy. Men were more strongly influenced by attitudes, whereas women were influenced more heavily by subjective norms and perceived behavioral control. Interestingly, subjective norms and perceived behavioral control had no significant impact on the decision regarding technology adoption among men at all. The gender differences were strengthened by older age [18]. Based on the TPB, the variables subjective norms and perceived behavioral control were included in the empirical study by analyzing participants’ perceived digital competence.

Based on the TRA [16,17], TAM is an applied and widely used model for describing and predicting the acceptance and use of a new information technology [12-14]. The TAM conceptualizes 2 central beliefs about a new technology that influence the intention to use it: perceived usefulness and perceived ease of use [14,19-22]. *Perceived usefulness* is defined as “the user’s perception of the degree to which using a particular system will improve his or her job performance” (eg, [22]), whereas *perceived ease of use* is defined as the “user’s perception of the extent to which using a particular system will be free of physical and mental effort” (eg, [22]). The TAM has been supported by many studies and has been applied in different contexts including the area of health information websites [23]. Various versions and extensions of the TAM have been developed. Bruner and Kumar [24] developed a “consumer technology acceptance model” (c-TAM) and demonstrated that in the consumer context, the fun of using a device was a more powerful predictor of attitude toward usage than the perceived usefulness of the device. Based on the TAM and its extension c-TAM, the variables perceived ease of use, perceived usefulness, and fun of use were included in the empirical study.

According to Dutta-Bergman [15], the functional theory of media use assumes that the use of a certain medium is motivated by different reasons and that communication behavior is goal-directed. In her study, motivation was a crucial factor in determining use of media (ie, the Internet). In her opinion, searching the Internet for health-oriented information is a reflection of health information orientation and is influenced by health consciousness and health awareness. There is relatively stable empirical evidence for a higher nutrition and health consciousness of women (eg, [25-33]). Therefore, the functional theory of media use lends support to the decision to focus on health and nutrition consciousness, as well as on motives for using the Internet as a source of health-related information and investigating the usage of different channels. All these variables were included in the empirical study.

Insights into gender differences in the virtual patient-physician relationship can also be drawn from the consumer behavior literature. According to Solomon [34], consumers’ reactions to stimuli depend on psychographic variables, which can be classified into activating, emotional, motivational, and cognitive processes, and they also depend on social influence variables (eg, normative and situational antecedents).

Based on the aforementioned concepts, the objectives of the paper are as follows:

1. Investigate differences in health-related information searching on the Internet in part 1 of the paper, especially by investigating gender differences in using the Internet for health-related information searching. This will be done by (1) analyzing gender differences in feelings toward the Internet and Web 2.0 for health-related information searching (emotional perspective); (2) analyzing gender differences in perceived behavioral control, which we conceptualize as perceived digital competence (cognitive perspective); (3) analyzing gender differences in the underlying motives for using the Internet for health-related information searching (motivational perspective); (4) analyzing gender differences in health and nutrition awareness (attitudinal perspective); (5) analyzing gender differences in the personal disposition of being well-informed as a patient (personal involvement perspective); and (6) analyzing gender differences in the importance of situational circumstances, which foster the usage of Internet health information searching as well as differences in the importance of normative pressure on the usage of the Internet for health-related information searching (situational involvement and/or a normative perspective).
2. Analyze gender differences in the present and future virtual patient-physician relationship in part 2 of the paper.

## Methods

### Participant Recruitment

An online survey of 1006 German patients was conducted in September 2012. The term “patients” in this paper refers to individuals who visited a physician at least once in the previous 3 months. The sample was drawn from an e-panel maintained by GfK HealthCare, a leading survey research company in Nuremberg, Germany. It was based on a randomly generated set of users who had visited a GP at least once during the 3 months before the beginning of the survey. Originally, 1561 individuals were contacted; 555 persons could not participate because they did not fulfill this criterion. The recruitment rate was 64.45% (1006/1561) [35]. In all, 20 participants were excluded from the analysis because of an extremely short answer time and/or inconsistent answer patterns (eg, flatliners, contradictions). Another 28 respondents were excluded because the number of missing values exceeded the limit of 30% in scale items [36]. The final sample consisted of 958 participants. Small monetary incentives were offered for survey completion.

### Questionnaire

The survey was designed by the researchers based on the existing literature and was guided by the research questions. All items apart from categorical variables (sociodemographic variables) and ordinal variables (frequency variables) were measured with 7-point rating scales. Most of the items had a “no answer” category as an alternative. Existing scales and items from the literature were used where applicable. Data were analyzed using SPSS version 22 (IBM Corp, Armonk, NY, USA).

### Measurement of Sociodemographic and Psychographic Variables

Multimedia Appendix 1 provides an extract of the questionnaire and refers to the corresponding literature for items. The original questionnaire was an online questionnaire in German; English translation is merely for the purpose of this paper. In the following section, the measurement of variables included in the present study will be explained. The denomination of items (F1_1 to F42_9, D1 to D8) in brackets refers to Multimedia Appendix 1.

#### Sociodemographic Variables

Age (D2_1), gender (D1), the highest educational level attained (D4), family status (D5), household size (D6_1), and the categorical monthly household net income were measured (D8).

### Part 1: Psychographic Variables

#### Feelings Toward the Internet and Other Web-Based Applications

Feelings toward the Internet and other Web-based applications in general were included in the questionnaire and measured by an item derived from Porter and Donthu [7,19] (F1_1).

#### Digital Literacy

Digital literacy is the ability to effectively and critically use a range of digital technologies. Literate individuals are able to make responsible choices and to access information and ideas in the digital world and to share information with others. In-line with previously published studies, digital literacy was measured with an item based on Norman and Skinner [7,8,37-39] (F2_1). In reference to the gender differences focus of this study, it has to be underlined that the construct digital literacy should be interpreted in the sense of perceived digital competence in order to do justice to the fact that especially in the area of technological knowledge it seems that women “are perhaps as susceptible to the belief in their own lack of technological ability as men are likely to delight in their own supposed superiority” [40]. Hence, our item measures perceived digital competence rather than real digital literacy.

#### Daily Internet Use

Respondents were asked about their daily Internet use, especially how many hours they spent on the Internet for private purposes on average on a daily, weekly, or monthly basis (total private use) (F3_1 to F3_3), and on average searching for health-related information (total private use for health-related information) (F4_1 to F4_3). We then calculated the total private Internet use and the total private Internet use for health-related information for each respondent on a daily basis.

#### Importance of Different Sources for Health-Related Information

For the purpose of this investigation, the importance of different sources (family, friends, physician, pharmacist, insurance agent, Internet, books/journals, other sources) was examined using items adopted from Moorman and Matulich [41] and Kummervold et al [42] (F6_1 to F6_8). The possible sources were listed in the questionnaire and the respondents had to rate the importance of each of the information sources.

#### Frequency of Using Different Channels on the Internet for Health-Related Information

For the purpose of investigating different search methods in the use of the Internet for health-related information, participants were asked to indicate how often they used the following channels on the Internet for health-related information searches: search engines (eg, Google), wikis (eg, Wikipedia), electronic databases and electronic papers as well as scientific papers and studies (eg, www.bmj.com), email, social networks/microblogs/networks (eg, Facebook), health forums (eg, www.imedo.de), podcasts (eg, YouTube), instant messaging/chat (eg, Skype, ICQ), and apps [43] (F7_1 to F7_10). Frequency was measured on a 6-point ordinal scale.

#### Motives of Using the Internet for Health-Related Information Searching

Concerning the motives of using the Internet for health-related information searching, different items from literature were used (F11_1 to F11_18). Perceived ease of use and perceived usefulness of the Internet to gain health-related information were measured by existing multi-item scales derived and adapted from Davis et al [44,45] and Venkatesh et al [9,13,46] and other authors investigating the motivational side of information searching [21,47-50]. Items measuring fun to use were adapted from Shih [51]. Additional items were developed after an extensive literature review in the health information search literature to measure the motives of saving time, of managing time flexibly, of the social component of sharing knowledge and/or making contact with someone easily, of being anonymous, and of being up-to-date.

#### Personal Disposition of Being Well-Informed as a Patient

According to Cacioppo and Petty [52] and Petty et al [53], the amount of information a person is seeking as well as the amount of cognitive effort and elaboration an individual is willing to devote to a specific task can be seen as individually varying personal disposition. In the area of health information searching, this means that some patients are inclined to prepare themselves for visiting a doctor and search for health-related information extensively, whereas others do this to a lesser extent [39]. Thus, some patients value health-related knowledgeability more highly because they may believe that being well-informed leads to better patient-physician communication or that the physician offers more time to well-informed patients. These individuals are more inclined to make significant health decisions on the basis of health-related information found on the Internet [7,53]. They even decide whether professional medical care is needed or not and alternatively rely on self-treatment based on their online findings [54]. For the purpose of investigating this personal disposition of being well-informed as a patient, a scale of 9 items (F20_1 to F20_9) was developed by the researchers. Some of the items were adapted from the health information orientation scale by Dutta-Bergman [15], from Simon et al [55], and from Wilson and Lankton [56].

#### Nutrition and Health Awareness and Attitude Toward Medical Support

Attitude is conceptualized by Solomon [34] as “a lasting, general evaluation of people (including oneself), objects, or issues” that merges into a system of values influencing the individual. The construct of health awareness primarily refers to the extent to which a person takes care of his/her own health [57-59]. We decided to denominate the construct health awareness instead of health consciousness because this sounds less clinical. Concerning nutrition and health awareness, 9 items were developed by the researchers based on a literature review and were partially adapted from the health consciousness attitude scale by Dutta-Bergman [15,60] and others [41,61] (F42_1 to F42_9).

### Part 1: Situational and Normative Influences on Health-Related Information Searching on the Internet

Eight additional items were developed and integrated into the questionnaire in recognition of the fact that using the Internet could not only be due to a reason lying in the respondent himself or herself, but rather because of normative or situational reasons (F12_1 to F12_8). Therefore, after literature reviews, some complementary items measuring situational and normative influences were derived and adapted from the TAM and the TPB [9,11,17,62-67] to represent these normative or situational reasons for using the Internet for health-related information searching.

### Part 2: Present and Future Communication With the General Practitioner and Internet-Based Treatment

For the purpose of investigating the present usage and future intention to communicate with the GP on the Internet and to partially replace personal communication with and treatment by a GP by the Internet, some additional items were developed by the researchers as shown in Multimedia Appendix 1. The frequency of using the Internet for communicating with the GP at present was measured by a single item on a 6-point ordinal scale (F13). Future intention to use the Internet for communicating with the GP was measured on a 7-point rating scale (F15_1). Additionally, the researchers measured which fields might conceivably be replaced by listing different areas of treatment along the virtual patient-physician relationship chain (F17_1 to F17_14). Finally, respondents were asked whether it was important to them to be able to use online treatments as well (F18_1) and how willing they would be to pay additionally for online treatment (F19_1).

## Results

### Sample Characteristics

A comparison of the sample used in the current study and German Internet users in 2012 (the German online population) [68] revealed that the sample represented the German online population quite well with regard to our most important variable gender (Table 1). Gender distribution in our sample (male: 54.0%, 517/958; female: 46.0%, 441/958) reflects the distribution among German Internet users (51.8% males, 48.2% females). Regarding age, participants in our sample were slightly older than those in the German online population. However, it should be noted that the comparable German Internet user basis were aged 10 years and older, whereas our study was based on respondents with a minimum age of 18 years. Another reason for this deviation probably lies in the selection criterion for participation; to qualify for our study, participants must have visited a GP at least once in the previous 3 months. With regard to education, the percentage of respondents with higher education was larger in our sample than in the German online population [68], which could be at least partially explained by the minimum age requirement of 18 years respectively the minimum age of 10 years in the comparison of the 2 databases. There were no comparable data in the German online population regarding marital status, household size, or household net income. Table 1 displays the characteristics of the sample.

**Table 1:** Characteristics of study sample compared to the German Internet population in 2012.

Variable and category		Female n=441	Male n=517	Total N=958	German Internet users^a^ N=57,045,000
**Gender, n (%)**					
	Men	0	517 (100.00)	517 (53.97)	29,553,000 (51.81)
	Women	441 (100.00)	0	441 (46.03)	27,492,000 (48.20)
**Age (years), mean (SD)**		41.21 (13.39)	45.88 (12.40)	43.73 (13.04)	
	Age range (years)	18-70	18-70	18-70	>10
**Age categories (years), n (%)**		441 (100.00)	517 (100.00)	958 (100.00)	
	<24	56 (12.70)	25 (4.84)	81 (8.45)	12,552,000 (22.00)
	25-44	192 (43.54)	198 (38.30)	390 (40.71)	20,344,000 (35.60)
	45-64	177 (40.14)	254 (49.13)	431 (44.99)	18,799,000 (32.96)
	>65	16 (3.64)	40 (7.74)	56 (5.85)	5,348,000 (9.38)
**Education, n (%)** ^b^		437 (100.00)	514 (100.00)	951 (100.00)	52,589,000 (100.00)^b^
	Without school qualification	2 (0.46)	2 (0.39)	4 (0.42)	
	Secondary general school	8 (1.83)	5 (0.97)	13 (1.37)	9,487,000 (18.04)^c^
	Polytechnic secondary school	43 (9.84)	77 (14.98)	120 (12.62)	
	Intermediate secondary school	142 (32.49)	127 (24.71)	269 (28.28)	29,467,000 (56.03)^d^
	Matura examination or higher	242 (55.38)	303 (58.95)	545 (57.31)	13,635,000 (25.93)^e^
**Number in household, n (%)**		439 (100.00)	517 (100.00)	956 (100.00)	
	1	90 (20.50)	117 (22.63)	207 (21.65)	
	2	169 (38.49)	194 (37.52)	363 (37.97)	
	3	87 (19.82)	113 (21.86)	200 (20.92)	
	4	83 (18.91)	72 (13.93)	155 (16.21)	
	>4	10 (2.28)	21 (4.06)	31 (3.24)	
**Marital status, n (%)**		439 (100.00)	509 (100.00)	948 (100.00)	
	Single	92 (20.95)	108 (21.22)	200 (21.10)	
	Close-partnered	110 (25.06)	105 (20.63)	215 (22.68)	
	Married	194 (44.19)	266 (52.26)	460 (48.52)	
	Divorced	36 (8.20)	28 (5.50)	64 (6.75)	
	Widowed	7 (1.60)	2 (0.39)	9 (0.95)	
**Monthly household net income (€), n (%)**		347 (100.00)	429 (100.00)	776 (100.00)	
	<1500	77 (22.19)	52 (12.12)	129 (16.63)	
	1500-2500	97 (27.95)	105 (24.47)	202 (26.03)	
	2501-3500	94 (27.09)	134(31.24)	228 (29.38)	
	3501-4500	53 (15.28)	68 (15.85)	121 (15.59)	
	>4500	26 (7.49)	70 (16.32)	96 (12.37)	

^a^ Rounded to 1000 people. Projected number of Germans who have used the Internet in the last 3 months. Age limit for questions concerning education and occupation: 16 years.

^b^ For the German Internet users, low education corresponds with levels 0, 1, and 2 of the ISCED classification system (up to secondary general school), medium education corresponds with levels 3 and 4 of the ISCED classification system (up to university entrance qualification), and high education corresponds with levels 5 and 6 of the ISCED classification system (higher than matura examination respectively university entrance qualification).

^c^ low education

^d^ medium education

^e^ high education

### Part 1: Health-Related Information Searching on the Internet

#### Gender Differences in Health Information Search Behavior on the Internet, Emotions, and Cognitions

Table 2 provides the corresponding results of unrelated *t* tests for the psychographic variables feelings toward the Internet and other Web-based applications, perceived digital competence, daily Internet use, importance of different sources for health-related information, and the frequency of using different search methods on the Internet for health-related information between men and women.

There was a significant difference between the 2 groups in terms of their perceived digital competence (*t*_899_=3.91, *P*<.001). Male respondents ascribed a higher level of perceived digital competence to themselves than female respondents did. When the participants were asked to evaluate the importance of different sources for health-related information, women rated friends (*t*_944_=–3.08, *P*=.002), books or journals (*t*_920_=–2.64, *P*=.009), the Internet (*t*_951_=–2.36, *P*=.02), and pharmacists (*t*_936_=–2.52, *P*=.012) more highly than men did (see Table 2 for details). The groups did not differ in their daily Internet use measured by the daily hours spent online for private use, or in their feelings toward the Internet and other Web-based applications in general. However, female respondents revealed a higher frequency of using the Internet for health-related information, but this difference did not meet statistical significance (*t*_572_=–1.76, *P*=.08). There were some differences between female and male respondents in the frequency of usage of different channels on the Internet for health-related information searches. Women reported a higher frequency of using health forums and blogs (Kendall’s tau-b=–0.06, *P*=.03). Women revealed a higher frequency of usage of search engines (eg, Google, Bing, or Yahoo!) for health-related information searching (Kendall’s tau-b=–0.06, *P*=.045). Men, on the other hand, revealed a higher frequency of using apps for health-related information searching (Kendall’s tau-b=0.07, *P*=.02).

To do justice to the relatively large sample size, which lead to a higher probability of differences becoming significant between the 2 groups, we added the effect size of Hedges’ *g* to evaluate the group differences in all the subsequent tables. The estimates of effect size can be used to determine the practical and/or theoretical relevance of an effect and the power of an analysis [69]. There are different ways to calculate effect sizes, the most often applied being Cohen’s *d* [69]. However, we decided to apply Hedges’ *g* [70-72]. While Cohen’s *d* favors identical sample sizes, Hedges’ *g* allows for different sample sizes, which we have in our study. In contrast to Cohen’s *d*, in Hedges’ *g* the population standard deviation is replaced by the pooled sample standard deviations, calculated by using a denominator of n-1 (see the detailed formula in Multimedia Appendix 2) [69,73,74]. All the differences in the following tables will be complemented by the report of Hedges’ *g*. We are aware of the potentially misleading influences of sample size and of the risk of overvaluing observed effects because of their significance [69]; therefore, we will interpret our results in the discussion section in the light of significance and magnitude of effect sizes [75].

Gender differences in the specified psychographic variables relating to health-related information searching are reported in the next section. Because of the large number of subsequent psychographic variables, we decided to summarize the motivational, attitudinal, and personal involvement items that might contribute to the explanation of gender differences in health-related information searching. Therefore, for each group of psychographic variables (motivational, attitudinal, and personal involvement processes underlying Internet health information searching) and the group of normative and situational influences, exploratory factor analyses (EFAs) were calculated for the total sample. Only those subsets of variables were factor analyzed, which were measured on an interval scale level (statistical precondition) and which could be assigned to a specific psychographic construct or to the group of normative and situational influences. This procedure was chosen to reduce the complexity versus the alternative of a large number of group differences on a single item level. The number of factors for each of the subscales was determined by the eigenvalue criterion; principal component analyses were used with a subsequent varimax rotation with Kaiser normalization. Items with low loadings and with loadings greater than 0.45 on more than 1 factor were removed. The variances extracted were reported only for the purified scales. The factor loadings of the purified scales were used for subsequent calculation of weighted means of factor sum scores. One advantage of this method is that items with the highest loadings on the factor have the largest effect on the factor score [76]. Afterwards, *t* tests were calculated for the weighted means of factor sum scores between male and female respondents and Hedges’ *g* scores were added. The differences are described in detail in the following section.

**Table 2:** Gender differences in Internet health information search behavior, emotions, and cognitions influencing Internet health-related information searching.

Variables		Female (n=441)		Male (n=517)		Total (N=958)		*t* (*df*)	Kendall’s tau-b	*P*	Hedges’ *g*
		n	Mean (SD) or median	n	Mean (SD) or median	n	Mean (SD) or median				
Feelings toward the Internet and other Web-based applications in general^a^		431	5.75 (1.04)	514	5.80 (1.16)	954	5.78 (1.11)	0.63 (943)		.53	0.05
Perceived digital competence^b^		441	5.72 (1.11)	517	5.99 (1.0)	958	5.87 (1.06)	3.91 (899)		<.001	0.26
**Daily Internet use (hours)**											
	Total private use	441	3.18 (2.52)	517	3.02 (2.07)	958	3.10 (2.29)	–1.05 (853)		.30	–0.07
	Total private use for health-related information	441	0.53 (2.05)	517	0.35 (0.86)	958	0.43 (1.53)	–1.76 (572)		.08	–0.12
**Importance of different sources for health-related information** ^c^											
	Family	437	4.85 (1.71)	511	4.85 (1.73)	948	4.85 (1.72)	0.04 (946)		.97	0.00
	Friends	436	4.36 (1.70)	510	4.01 (1.74)	946	4.17 (1.73)	–3.08 (944)		.002	–0.20
	Physician	440	6.44 (0.90)	515	6.41 (0.98)	955	6.42 (0.95)	–0.40 (953)		.69	0.03
	Pharmacist	432	5.15 (1.54)	506	4.89 (1.59)	938	5.01 (1.57)	–2.52 (936)		.012	–0.17
	Insurance agent	405	1.75 (1.34)	486	1.80 (1.34)	891	1.78 (1.34)	0.63 (889)		.53	0.04
	Internet	437	4.73 (1.44)	516	4.51 (1.43)	953	4.61 (1.44)	–2.36 (951)		.02	–0.15
	Books/journals	425	4.44 (1.64)	497	4.15 (1.70)	922	4.29 (1.68)	–2.64 (920)		.009	–0.17
	Other sources	280	3.02 (1.8)	352	2.81 (1.79)	632	2.90 (1.79)	–1.5 (630)		.14	–0.12
**Frequency of usage of different channels on the Internet for health-related information searches, median** ^d^											
	Search engines	441	3	517	4	958			–0.06	.045	
	Wikis online encyclopedia	441	4	517	4	958			–0.02	.41	
	Electronic databases/journals	441	5	517	5	958			0.03	.39	
	Email	441	5	517	5	958			0.03	.38	
	Social network/microblogging	441	6	517	6	958			–0.03	.27	
	Health forums/blogs	441	5	517	5	958			–0.06	.03	
	Podcasts	441	6	517	6	958			–0.03	.35	
	Videoconferences	441	6	517	6	958			0.02	.55	
	Instant messaging/chat	441	6	517	6	958			–0.04	.24	
	Apps	441	6	517	6	958			0.07	.02	

^a^ 1=very negative, 7=very positive.

^b^ 1=not literate at all, 7=very literate.

^c^ 1=not important at all, 7=very important.

^d^ 1=daily, 2=weekly, 3=less often than weekly, 4=monthly, 5=less often than monthly, 6=never.

#### Gender Differences of Weighted Means of Factor Sum Scores for Motives Influencing Internet Health Information Searching: Exploratory Factor Analysis 1

Strong evidence was found for the existence of different motives when using the Internet for health-related information searching. Because the same procedure for the EFA was executed for all the groups of variables (attitudinal, personal involvement, situational, and normative perspective), it is only described in detail for the EFA 1. Detailed information for the other EFAs are included in the respective tables in Multimedia Appendix 2. An EFA of the 18 items measuring the underlying motives for Internet health information searching lead to a 3-factor solution of the purified scale explaining 66.69% of variance (2 items were excluded from further analysis due to low factor loadings.). As required, the Kaiser-Meyer-Olkin (KMO) measure of the appropriateness of the sample was not significant (*P*=.93) and the Bartlett-Test of sphericity was significant (χ^2^_120_=8345.2, *P*<.001). The reduced scale lead to a 3-factor solution for the motivational variables underlying Internet health information searches. The first factor (eigenvalue=7.28) consisted of 7 items featuring the social motive and enjoyment of Internet health information searching, the second factor (eigenvalue=2.38) comprised 6 items representing perceived usefulness of the Internet as a medium for health information searching, and the third factor (eigenvalue=1.01) was construed by 3 items focusing on the usefulness of the information gained from the Internet for health information searching. Table A in Multimedia Appendix 2 shows the fully rotated factor component matrix. For all the remaining 16 variables, 3 weighted means of factor sum scores were calculated (see Table E in Multimedia Appendix 2 for details of the formula) and *t* tests were calculated (see Table 3).

Women used the Internet to a greater extent than men did due to a social motive and enjoyment of Internet health information searching (*t*_835_=–2.31, *P*=.02). Additionally, women judged the usefulness of the information gained from the Internet health information searching more highly than men did (*t*_943_=–3.16, *P*=.002). There was a difference between men and women according to the perceived usefulness of the Internet as a medium for health information searching, but these differences did not meet statistical significance (*t*_908.55_=–1.94, *P*=.05).

**Table 3:** Gender differences of weighted means of factor sum scores for motives influencing Internet health information searching on an aggregate level.

Factors	Female (n=441)		Male (n=517)		Total (N=958)		*t* (*df*)	*P*	Hedges’ *g*
	n	Mean (SD)	n	Mean (SD)	n	Mean (SD)			
Social motive and joyousness of Internet health information searching	387	4.50 (1.50)	450	4.27 (1.48)	837	4.37 (1.49)	–2.31 (835)	.02	–0.15
Perceived usefulness of the Internet for health information searching	417	6.08 (0.93)	494	5.94 (1.12)	911	6.0 (1.04)	–1.94 (908.55)	.05	–0.14
Usefulness of the information gained from Internet health information searching	434	5.37 (1.17)	510	5.12 (1.26)	944	5.23 (1.22)	–3.16 (942)	.002	–0.21

#### Gender Differences of Weighted Means of Factor Sum Scores for Attitudes Influencing Internet Health Information Searching: Exploratory Factor Analysis 2

An EFA of the 9 items measuring the attitudinal influences deriving from different health and nutrition awareness and proneness to use medical support lead to a 2-factor solution for the purified scale explaining 61.14% of variance (see Table B in Multimedia Appendix 2 for details of the analysis). For all the remaining 6 variables, 2 weighted means of factor sum scores were calculated (see Table E in Multimedia Appendix 2 for details of the formula) and *t* tests were executed between the 2 weighted means of factor sum scores of the subsamples of female and male respondents.

As is shown in Table 4, there were significant differences in both areas between female and male respondents. Women had higher health and nutrition awareness on an aggregate level than men (*t*_953_=–3.07, *P*=.002) and a greater reluctance to make use of medical support (*t*_951_=–2.58, *P*=.01).

**Table 4:** Gender differences of weighted means of factor sum scores for attitudes influencing Internet health information searching on an aggregate level.

Factors	Female (n=441)		Male (n=517)		Total (N=899)		*t* (*df*)	*P*	Hedges’ *g*
	n	Mean (SD)	n	Mean (SD)	n	Mean (SD)			
Health and nutrition awareness	440	5.24 (1.08)	515	5.02 (1.15)	955	5.12 (1.12)	–3.07 (953)	.002	–0.20
Reluctance to make use of medical support	439	4.79 (1.56)	514	4.52 (1.52)	953	4.64 (1.54)	–2.58 (951)	.010	–0.18

#### Gender Differences of Weighted Factor Sum Scores for the Personal Disposition of Being Well-Informed as a Patient: Exploratory Factor Analysis 3

An EFA of the 9 items measuring the personal disposition of being well-informed as a patient lead to a single factor solution explaining 52.93% of variance (see Table C of Multimedia Appendix 2 for details of the analysis). For all 9 variables, 1 weighted factor sum score was calculated (see Table E in Multimedia Appendix 2 for details of the formula) and a *t* test for the weighted mean of the factor sum score was calculated between the 2 subsamples of female and male respondents. There was no significant difference in this single factor of personal disposition of being well-informed as a patient in general. Therefore, it was decided to investigate the single items too (see Table 5).

**Table 5:** Gender differences of weighted factor sum scores for the personal disposition of being well-informed influencing Internet health information search behavior on an aggregate as well as on a basis level.

Factors/Variables		Female (n=441)		Male (n=517)		Total (N=958)		*t* (*df*)	*P*	Hedges’ *g*
		n	Mean (SD)	n	Mean (SD)	n	Mean (SD)			
Personal disposition of being well-informed as a patient		413	4.05 (1.41)	486	3.94 (1.37)	899	3.99 (1.39)	–1.19 (897)	.24	–0.08
**Different aspects of the personal disposition of being well-informed as a patient** ^a^										
	It is important to me to be well-informed when consulting a physician.	436	4.70 (1.70)	514	4.72 (1.72)	955	4.71 (1.71)	0.12 (953)	.91	0.01
	When I obtain health-related information from the Internet, I need to talk about this information with my physician.	436	3.99 (1.90)	514	4.12 (1.82)	950	4.06 (1.86)	1.11 (948)	.27	0.07
	When a therapy is prescribed for me, I look for alternative therapies on the Internet.	439	4.30 (1.92)	514	4.33 (1.85)	953	4.32 (1.88)	0.23 (951)	.82	0.02
	Sometimes I have the feeling that I am better informed about my medical condition than my physician.	439	3.78 (2.01)	513	3.55 (1.92)	952	3.66 (1.96)	–1.79 (950)	.08	–0.12
	If the patient is informed, the communication with the physician is improved.	437	4.74 (1.70)	510	4.68 (1.72)	947	4.71 (1.71)	–0.51 (945)	.61	–0.04
	I only decide whether a consultation with a physician is really necessary, once I have conducted some health information searches on the Internet.	437	3.18 (1.97)	512	2.93 (1.88)	949	3.05 (1.92)	–1.98 (947)	.048	–0.13
	If some medicines have been prescribed, I look for information about them on the Internet.	440	4.25 (2.04)	515	3.99 (2.01)	955	4.11 (2.03)	–2.01 (953)	.045	–0.13
	If the patient is informed, the physician allows more time for the treatment.	427	3.32 (1.92)	502	3.42 (1.88)	929	3.37 (1.90)	0.75 (927)	.46	0.05
	The physician is more likely to prescribe a requested medicine, if the patient is informed.	418	3.66 (1.98)	500	3.58 (1.92)	918	3.61 (1.95)	–0.62 (916)	.54	–0.04

^a^ 1=strongly disagree, 7=strongly agree.

As shown in Table 5, women actually differed to a certain extent in this facet of personality, but only in some distinctive aspects. Women seemed to decide on the basis of Internet health information whether to consult a physician or not to a greater extent than men did (*t*_947_=–1.98, *P*=.048) and they seemed to inform themselves more than men about suggested remedies on the Internet (*t*_953_=–2.01, *P*=.045). Additionally, female patients sometimes felt better informed about their medical state than their physician to a greater degree in comparison to male patients, but the difference did not meet statistical significance (*t*_950_=–1.79, *P*=.08).

#### Gender Differences of Weighted Means of Factor Sum Scores for Situational and Normative Variables Influencing Internet Health Information Searching Exploratory Factor Analysis 4

An EFA of the 5 items measuring the underlying situational and involvement influences on Internet health information searching lead to a 2-factor solution explaining 78.88% of variance (see Table D in Multimedia Appendix 2 for details of the analysis). For all 5 variables, 2 weighted means of factor sum scores were calculated (see Table E in Multimedia Appendix 2 for details of the formula) and *t* tests were executed (see Table 6).

**Table 6:** Gender differences of weighted means of factor sum scores for situational and normative variables influencing Internet health information searching on an aggregate level.

Factors	Female (n=441)		Male (n=517)		Total (N=958)		*t* (*df*)	*P*	Hedges’ *g*
	n	Mean (SD)	n	Mean (SD)	n	Mean (SD)			
Situational influences on Internet health information searching	434	5.54 (1.23)	512	5.35 (1.27)	946	5.44 (1.25)	–2.25 (944)	.03	–0.15
Normative influences on Internet health information searching	384	3.48 (1.88)	460	3.33 (1.85)	844	3.40 (1.86)	–1.20 (842)	.23	–0.08

Women seemed to be caught in a crossfire of situational, but not normative influences, to a greater extent than men which reinforced the usage of the Internet for health-related information searches. The factor including situational influences had a higher mean score for women than for men (*t*_944_=–2.25, *P*=.03). The most striking result to emerge from the data was that neither women nor men were exposed to a normative influence when using the Internet or Web 2.0 for health-related information searching. The mean was comparably low for both groups (see Table 6). There was no difference between the 2 subgroups in reference to the normative influence of important individuals or individuals whom the respondents looked up to who might recommend the usage of the Internet for health-related information searching. Comparing the results between women and men, situational influences were predominantly important for women, and to a lesser extent for men, whenever they used the Internet for health-related information searching.

### Part 2: The Virtual Patient-Physician Relationship

#### Gender Differences in Present Communication With the General Practitioner on the Internet

For the purpose of establishing whether there are gender differences in the present virtual patient-physician relationship, several unrelated *t* tests and Kendall’s tau-b tests were executed. In reference to the actual use of the Internet for communicating with the GP at present, there were significant differences between the 2 groups. Given that respondents were asked about the frequency of present use of online communication with the GP on an ordinal scale (1=daily, 6=never), Kendall’s tau-b was calculated to investigate gender differences. Men reported a higher frequency of communicating online with the GP than women at present (Kendall’s tau-b=0.07, *P*=.02).

#### Gender Differences in Future Intention to Replace Personal Communication With the General Practitioner and Treatment by the Internet

In reference to the future behavioral intention of using the Internet for communication with the GP, male respondents were more prone to replace personal communication with the GP and treatment by the Internet (see Table 7). Male respondents had a higher intention to use the Internet for communicating with the GP in general than female respondents did (*t*_905_=4.15, *P*<.001) and they were more ready to pay additionally for online treatment (*t*_941_=2.24, *P*=.03). Gender differences were found with regard to the importance of being able to additionally use online treatment, but this did not meet statistical significance (*t*_946_=1.88, *P*=.06). To see if the subsamples categorized possible areas of the physician-patient relationship in reference to their imagination of being replaced by the Internet differently, additional unrelated *t* tests were executed for each of the listed areas in the questionnaire (see Multimedia Appendix 1). As shown in Table 7, men had a higher intention to replace personal communication with the GP by the Internet for the fixing of personal appointments (*t*_841_=2.13, *P*=.03), the supervision of chronically ill people (*t*_943_=2.45, *P*=.01), and for routine treatments (sore throat, head cold, etc) (*t*_944_=2.45, *P*=.01) than women did. Gender differences with regard to the discussion of critical test results were found, but did not meet statistical significance (*t*_947_=1.85, *P*=.07). By looking at the ranking of the means of the total sample and the 2 subsamples (see Table 7), the following aspects of the virtual physician-patient relationship were the most conceivable in terms of being replaced by the Internet in the future: (1) fixing of personal appointments (female: mean 6.21, SD 1.56; male: mean 6.41, SD 1.26), (2) referrals to other doctors (female: mean 5.99, SD 1.66; male: mean 5.86, SD 1.69), (3) writing of prescriptions (female: mean 5.60, SD 1.97; male: mean 5.68, SD 1.83), (4) discussion of normal results of a test (female: mean 5.07, SD 2.17; male: mean 4.95, SD 2.15), and (5) secondary effects of drugs (female: mean 4.74, SD 2.14; male: mean 5.00, SD 2.00).

**Table 7:** Gender differences for future intention to replace personal communication and treatment by the Internet.

Variables		Female (n=441)		Male (n=517)		Total (N=958)		*t* (*df*)	*P*	Hedges’ *g*
		n	Mean (SD)	n	Mean (SD)	n	Mean (SD)			
Intention of using the Internet more often in the future for communicating with the GP^a^		438	4.05 (2.31)	513	4.66 (2.17)	951	4.38 (2.54)	4.15 (905)	<.001	0.27
Importance of being able to use online treatment as well^b^		436	3.47 (1.99)	512	3.71 (2.04)	948	3.60 (2.02)	1.88 (946)	.06	0.12
Willingness to pay a certain amount additionally for online-treatment^c^		436	2.15 (1.74)	515	2.42 (1.87)	951	2.30 (1.81)	2.24 (941)	.03	0.15
**For which of the following areas could you imagine the replacement of personal communication with your GP through Internet communication in the future?** ^a^										
	Fixing of personal appointments	440	6.21 (1.56)	514	6.41 (1.26)	954	6.32 (1.41)	2.13 (841)	.03	0.14
	Preliminary advice	436	4.75 (2.19)	511	4.86 (2.10)	947	4.81 (2.14)	0.80 (945)	.42	0.05
	Writing of prescriptions	439	5.60 (1.97)	512	5.68 (1.83)	951	5.64 (1.90)	0.60 (902)	.55	0.04
	Doctor’s notes/certificates of health	434	4.58 (2.32)	504	4.67 (2.27)	938	4.63 (2.29)	0.62 (936)	.54	0.04
	Referrals to other doctors	438	5.99 (1.66)	513	5.86 (1.69)	951	5.92 (1.68)	–1.17 (949)	.24	–0.08
	Discussion of “normal” test results	437	5.07 (2.17)	514	4.95 (2.15)	951	5.01 (2.15)	–0.87 (949)	.39	–0.06
	Discussion of “critical” test results	437	2.61 (2.01)	512	2.85 (2.05)	949	2.74 (2.04)	1.85 (947)	.07	0.12
	Follow-up checks after treatment	436	3.13 (2.13)	506	3.29 (2.05)	942	3.21 (2.09)	1.21 (940)	.23	0.08
	Supervision of chronically ill people	435	3.90 (2.16)	510	4.25 (2.17)	945	4.09 (2.17)	2.45 (943)	.01	0.16
	Secondary effects of drugs	438	4.74 (2.14)	512	5.00 (2.00)	950	4.88 (2.07)	1.95 (902)	.052	0.13
	Routine treatments (eg, sore throat, head cold)	436	3.96 (2.25)	510	4.31 (2.15)	946	4.15 (2.20)	2.45 (944)	.014	0.16
	Psychotherapy	435	2.42 (2.00)	505	2.48 (1.95)	940	2.45 (1.97)	0.50 (938)	.62	0.03
	Mental health problems	438	2.59 (2.03)	504	2.74 (1.95)	942	2.67 (1.99)	1.21 (940)	.23	0.08
	Acute disorders (eg, chest pains)	438	2.42 (2.04)	505	2.56 (2.01)	941	2.50 (2.03)	1.11 (939)	.27	0.07

^a^ 1=highly unlikely, 7=very likely.

^b^ 1=not important at all, 7=very important.

^c^ 1=I would not be willing at all, 7=I would be willing.

## Discussion

### Principal Findings

In reviewing the literature, only scarce empirical evidence was found on the underlying emotional, motivational, normative and situational, attitudinal, cognitive, and personal involvement variables, which may explain gender differences in Internet health-related information searching and on gender differences in the virtual patient-physician relationship. Therefore, the aim of the current investigation was to shed light on gender differences in these areas.

In order to do justice to the large sample size, we added the effect size Hedge’s *g* for all *t* test values in the Results section. According to Cohen [77,78], a measure of 0.2 reflects a small effect, 0.5 reflects a medium effect, and a score greater than 0.8 reflects a large effect. Bortz and Döring [79] classify effect sizes greater than 0.50 as large, effect sizes between 0.50 and 0.30 as medium, effect sizes between 0.30 and 0.10 as small, and those less than 0.10 as trivial, the latter indicating low practical relevance. However, according to Fröhlich et al [74,80], effect sizes have to be specified according to the research field and should be interpreted dynamically (ie, in the light of the methods applied or in comparison to other extant results reported in similar research). The design of the study may also influence effect size [75]. From the point of view of effect sizes, experimentation is desirable because of the possibility of causality inference and because effect sizes seem to be more accurate. According to McCartney and Rosenthal [75], experiments in the field are likely to cause larger effects, whereas effect sizes from nonrandomized and quasi-experimental designs are likely to be affected by possible confounding variables that may interfere with the interesting variables.

The effect sizes in our study are mostly small, but exceeded the limit of 0.1 as suggested by Bortz and Döring [79] in most cases. However, we did not manipulate conditions or interventions to investigate gender differences in an experimental setting, but investigated gender differences in a real field research setting on an exploratory basis. In addition, to the best of our knowledge, comparable reports of measures of effect sizes in the literature in the area of gender differences in health-related information search behavior and the virtual patient-physician relationship are lacking, further obstructing the comparison of our effect sizes against other research findings. In reference to McCartney and Rosenthal [75], “no criterion can be developed to separate small, useless effects from small, useful ones; researchers need to evaluate effect sizes using logic and argument.” Therefore, we discuss our results with the gender differences and the effect sizes in the light of the exploratory nature of our study.

### Part 1: Health-Related Information Searching on the Internet

In reference to behavioral variables the study is by trend in-line with studies reporting that women are more frequent users of the Internet for health-related information searches [2-5,8,48], but the respective gender differences found in our study did not meet statistical significance. Additionally, it was demonstrated that women and men differ in their frequency of usage of different channels on the Internet for health-related information searching. In comparison to men, women report a higher frequency of using health forums and blogs and search engines (according to Kendall’s tau-b test) as well as search engines, but the latter does not meet statistical significance. Friends, pharmacists, books and journals, and the Internet are more important sources for health-related information searching for women than for men. Male respondents, conversely, use apps more often than women for health-related information searching. This is in-line with research demonstrating that men consistently show higher levels of mobile Internet and app usage than women do (eg, [81]). For instance, the German Digitalbarometer, a telephone survey conducted 2012 in cooperation between TNS Emnid, IP Deutschland, and the trade magazine *Werben & Verkaufen* among 1142 Germans between 14 and 64 years [82] reported that 36% of men and 18% of women used apps. One important explanation for the higher usage of mobile devices and apps by men is given by Or and Karsh [83], who report that women have higher computer anxiety and less perceived behavior control. This argument is in-line with the fact that, in our study, men ascribe themselves higher perceived digital competence (cognitive perspective)*.* Technological competence refers to sexual identity and Cockburn argues that femininity seems to be incompatible with technological competence and women who feel technologically competent perceive themselves as being more manly [40]. Therefore, being comfortable with technology contributes more or less to some kind of male gender identity [40,84]. However, men ascribing themselves higher perceived digital competence may not correspond to real differences in digital literacy because differences were not measured by observation, but were based on self-reported answers. Differences in self-ascribed digital competence may simply reflect differences in culturally evolved gender identity. Nevertheless, a higher perceived digital competence may also prevent computer anxiety and may correspond with higher behavior control in the area of Internet information searching.

The current study found that there are no differences between the female and male respondents in their feelings toward the Internet and other Web-based apps in general.

The next question in this research was whether women and men differ in their motivations to use the Internet for health-related information searching. The most interesting finding was that women use the Internet for health-related information searching to a higher degree than men for social reasons and for pleasure. They evaluate it as a more useful medium and they perceive the gained information as more useful than men do. When looking at the differences on the level of the items, the Internet is attractive for women because it is an efficient method of searching (easy, quick, always available, capable of enhancing search success) because of its social dimensions (offering different formats, getting in contact with other people easily) and its entertainment potential. These results can be explained from a social role perspective. Due to the multitasking agenda of women, especially those of middle age, who play key roles as health managers and family caregivers [1,85,86], efficiency is very important. On the other hand, the Internet offers a new way of getting in contact with other people at times when the children are asleep, for example. Therefore, when women are responsible for young children, they have to overcome more obstacles when they want to meet other people in person. Thus, the social dimensions of the Internet may be more attractive for women than for men and the entertaining dimensions of the Internet may be of higher importance for them than for men.

With regard to the question of how situational involvement differs between women and men in relation to health-related information searching on the Internet, this study found that situational influences are predominantly important for women, and to a smaller extent for men, whenever they use the Internet for health-related information searching. Surprisingly, normative influences seem to make no contribution to gender differences in usage of the Internet for health-related information searching. A possible explanation for this might be that women, especially middle-aged women, sometimes work part-time because of their manifold roles and therefore have only limited access to and limited time for the Internet. This may cause a higher dependency on situational circumstances and a higher situational involvement with the Internet and Web 2.0. Nevertheless, this explanation must be interpreted with caution, because there are many middle-aged women who work full time in spite of possible manifold roles. Therefore, this interpretation cannot be extrapolated to all women; hence, there is room for many other complementary root cause analyses.

From an attitudinal perspective, the results are consistent with those of other studies revealing that women show higher nutrition and health awareness across different countries and settings (eg, [28-33]) and prefer homeopathic remedies to a higher extent, which was also found consistently in studies from different countries (eg, [87,88]). However, the findings of this study do not support the results from a recent study by Cho et al [57]; they found that men had higher health consciousness.

This study found that women are more reluctant to visit a physician than men. This result is contrary to a recent study from Smith et al [29], who found that men have a higher reluctance than women to visit a doctor for minor mental health concerns, but seem to seek help once a problem reaches a specific threshold. In our study, the items were formulated in a more general manner and did not focus on mental health problems. Therefore, our results in this context may be explained partially by the personal disposition of being well-informed as a patient, which is higher for women than for men. Women may often decide to visit a physician only once they have conducted some Internet health information searches. They are also more prone to look for information about prescribed remedies. Altogether, they seem to value being well-informed as a patient more highly than men and they strive to be better informed through the search for health-related information on the Internet. Thus, because of their need to be well-informed about their symptoms, they hesitate to consult a GP more than men in the case of illness. However, social role interpretations are not only useful in explaining the frequency differences between men and women in Internet health information searches, but also in explaining the underlying motives and attitudes toward Internet health information searching.

### Part 2: The Virtual Patient-Physician Relationship

At present, men report a higher frequency of communicating online with the GP and they are also more willing than women are to replace personal communication with the GP and treatment by the Internet in the future. Men can imagine fostering the virtual patient-physician relationship in the areas of making personal appointments, the supervision of chronically ill people, and for routine treatments (eg, sore throat, head cold). Additionally, they are more willing to pay a certain amount of extra money for online treatment. We see 2 main explanations for these findings. First, and as outlined previously, women perceive themselves as less digitally literate than men and, therefore, may feel a higher level of unease with regard to replacing the relatively intimate personal face-to-face GP consultation by a virtual one, which is probably rated as being less intimate. Secondly, from a social role perspective, women visit GPs not only for themselves, but also in their role as caregiver to their children. Hence, the replacement of a personal consultation by a virtual consultation may be perceived as being even more difficult if women are acting on behalf of someone else, especially their own children.

Hence, the replacement of the personal dimension through the Internet may be more difficult for women than it is for men. Reduced willingness to pay additionally for online treatment may also be explained by women’s smaller amount of disposable income. Comparing the household net income of the female and the male subsample, in-line with the census data, it was shown that the household net income was higher for the male subsample. Therefore, it may be more affordable for men to pay a certain amount of extra money for online treatment.

### Limitations

The study is not without limitations. There is the possibility of selection bias among respondents, although random selection out of the database was held to minimize its likelihood. The recruitment rate of 64% for this online panel sample also indicates that selection bias among respondents is probably low. A demographic comparison showed that our sample reflects the German online population relatively well. However, in the subsample of male respondents, the age category of older men (45 years and older) was overrepresented and there were also more respondents with higher education than in the general online population for both of the subgroups. Future studies may try to make use of a larger randomized sample of the average online population.

The questionnaire was very comprehensive because of the many variables that were addressed, which might raise the issue of fatigue among the respondents. However, the exact duration of the survey completion was automatically measured and saved in a control variable offering the possibility to control for answer duration and to exclude participants with an extremely short answer time from the analysis. In addition, data were also analyzed for inconsistent answer patterns (eg, flatliners, contradictions). Several multi-item scales were aggregated using EFAs. However, such data treatment for the sake of complexity reduction always leads to a loss of variance of the individual items. Our measurement of daily Internet use by asking respondents for their average usage may have been challenging for participants, especially for individuals with an intermittent usage pattern. An alternative would have been to ask respondents for their duration of Internet usage in the previous week (or month). However, such alternative measurement faces the problem that the previous week (month) might not be representative of the average duration. The construct digital literacy may face a special problem for a gender-specific research focus. The problem is that men and women perceive digital competence differently with men being, in general, more self-confident in this area and women facing less self-ascribed digital affinity. These interpretations may follow differences in self-identity as has been elaborated previously. For this reason, the results conveying gender differences for the construct digital literacy were interpreted as differences in perceived digital competence from a gender identity perspective.

Our study can be categorized as being exploratory in nature, delivering some pioneer knowledge in investigating reasons for gender differences in health-related information search behavior and the virtual patient-physician relationship. Although the *t* tests and Kendall’s tau-b tests demonstrated significant differences in many areas, the effect sizes (Hedges’ *g*) were relatively low (however, low effect sizes are not necessarily a limitation). It seems possible that the small effect sizes may be traced back to the field research paradigm instead of experimental design. Nevertheless, due to the exploratory nature of the study, we think that the results deliver interesting insights into gender differences in health-related information search behavior and the underlying psychographic, situational, and normative variables. Results also shed light on the virtual patient-physician relationship.

Another limitation of our study is that gender differences are likely to be bounded to the respective cultural background, especially when they are interpreted from a social role perspective. Although we believe that the findings are generalizable beyond the German population to a certain extent (eg, to other German-speaking countries), comparable studies in other countries would bring forward the generalizability of our results.

It would also be interesting to investigate the research questions and validate our results on gender differences by using other methods of inquiry, samples, and countries in the future.

### Practical Implications

The first implication that can be derived from our study is one from a more general gender perspective. Results from this survey are mostly in-line with previous studies demonstrating that women ascribe themselves a lower degree of digital competence than men. The current study delivers an additional argument from the health sector, namely that the government might want to be more proactive in enabling and encouraging women to be interested in technology and in technical devices from an early age.

Our study delivered the interesting finding that women have a higher social motive for health-related information searches and value the enjoyment of Internet health information searching to a higher degree than men do. Hence, measures to increase the pleasure of health information searching may be especially beneficial to women. This may be interesting for government institutions (eg, for health consciousness campaigns), but it is also of interest to the pharmaceutical industry wanting to promote their products. For instance, advergames targeted at female virtual players could be a means to reinforce health consciousness (educational advergames) or brand knowledge and brand awareness of pharmaceutical products or dietary supplements [89].

The lower health and nutrition awareness of men could be interesting for GPs, for the government, for the insurance industry, and for entrepreneurs developing apps. Men have a shorter life expectancy, which may be influenced to a certain degree by their lower health and nutrition awareness. Because men have a higher tendency to use apps for health-related information searching, men could be an interesting target group for health-promoting apps and/or fitness apps, which have been booming in recent years. These apps could also be interesting for the insurance industry and the government, which is confronted with ever-increasing expenditures in the health sector.

The fact that men are also more interested in fostering the virtual patient-physician relationship may be of special interest for GPs. For example, if a GP wants to reduce waiting times and operate more efficiently (eg, through Internet communication for administrative purposes), men may be more easily convinced than women.

Aside from gender, there are several areas for GPs in which the virtual patient-physician relationship could be reinforced: the fixing of personal appointments, referrals to other doctors, the writing of prescriptions, discussions of normal test results, and doctor’s notes/certificates of health. If a GP intends to foster her/his customer orientation, she/he may think about reducing waiting times by offering more online services in the preceding areas. An important step here would be to clarify the legal framework conditions for implementing an enhancement of the virtual patient-physician relationship. Yet it will be necessary to segment the patient base according to their individual disposition toward fostering the virtual patient-physician relationship, which may be influenced by gender.

## Appendix

Multimedia Appendix 1 Extract of the questionnaire and justification of items.

Multimedia Appendix 2 Additional tables (A-F) and methodological details of exploratory factor analyses 1- 4.

## Abbreviations

EFA: exploratory factor analysis
GfK: Gesellschaft für Konsumforschung
GP: general practitioner
TAM: technology acceptance model
TPB: theory of planned behavior
TRA: theory of reasoned action
