# Supplementary material for: Gender Differences in Searching for Health Information on the Internet and the Virtual Patient-Physician Relationship in Germany: Exploratory Results on How Men and Women Differ and Why
Source: J Med Internet Res. 2015 Jun 22;17(6):e156. doi: 10.2196/jmir.4127 (PMC4526954; doi:10.2196/jmir.4127)
Supplement: Multimedia Appendix 1 [file jmir_v17i6e156_app1.pdf]

## MULTIMEDIA APPENDIX 1 - Questions and Justification of Items
## (Original Language and Translation for the Paper)

### ALLGEMEINE EINSTELLUNG GEGENÜBER DEM INTERNET / GENERAL ATTITUDE TOWARD THE INTERNET

F1_1 **1. Welche Gefühle haben Sie gegenüber dem Internet und anderen webbasierten Anwendungen (z.B. Anwendungen am Smartphone oder Tablet-PC) im Allgemeinen?**

**In general, what kind of feelings do you have toward the Internet and other web-based applications (e.g. apps on the smart phone or tablet)?**

Adopted from [7,19]

| | Very negative | | | | | | Very positive | No answer |
|---|---|---|---|---|---|---|---|---|
| F1_1 | 1 | 2 | 3 | 4 | 5 | 6 | 7 | 8 |

### DIGITAL LITERACY

F2_1 **2. Wie würden Sie sich auf einer Skala von 1 (Ich kenne mich überhaupt nicht aus) bis 7 (Ich kenne mich sehr gut aus), im Umgang mit dem Internet im Allgemeinen, selbst einstufen?**

**How would you rate your own Internet skills on a scale from 1 (I am not literate at all) to 7 (I am very literate)?**

Adopted from [7,38,39]

| | Not literate at all | | | | | | Very literate | No answer |
|---|---|---|---|---|---|---|---|---|
| F2_1 | 1 | 2 | 3 | 4 | 5 | 6 | 7 | 8 |

### NUTZUNGSVERHALTEN/USAGE BEHAVIOR

**3. Wenn Sie an Ihre Internetnutzung denken, wie viele Stunden nutzen Sie durchschnittlich das Internet für private Tätigkeiten?** (Bitte wählen Sie eine Antwortoption (pro Tag oder pro Woche oder pro Monat) aus, die Ihrer Nutzung am ehesten entspricht)

F3_1 Durchschnittliche Internetnutzung in **Stunden pro Tag** __________
F3_2 Durchschnittliche Internetnutzung in **Stunden pro Woche** __________
F3_3 Durchschnittliche Internetnutzung in **Stunden pro Monat** __________

**If you think about your Internet usage, how many hours do you spend on the Internet on average for private purposes?** (Please select one answer option (per day or per week or per month), which corresponds to your usage most closely)

F3_1 Average Internet usage **in hours per day** __________
F3_2 Average Internet usage **in hours per week** __________
F3_3 Average Internet usage **in hours per month** __________

Adopted from [7]

**4. Wie viele Stunden nutzen Sie durchschnittlich das Internet für die Suche nach gesundheitsorientierten Informationen?**
(Bitte wählen Sie eine Antwortoption (pro Tag oder pro Woche oder pro Monat) aus, die Ihrer Nutzung am ehesten entspricht)

F4_1 Durchschnittliche Internetnutzung in **Stunden pro Tag** __________
F4_2 Durchschnittliche Internetnutzung in **Stunden pro Woche** __________
F4_3 Durchschnittliche Internetnutzung in **Stunden pro Monat** __________

**How many hours on average do you spend on the Internet on average searching for health-related information?**

Adopted from [7]

F4_1 Average Internet usage **in hours per day** ___________

F4_2 Average Internet usage **in hours per week** ___________

F4_3 Average Internet usage **in hours per month** ___________

**5. Welche Bedeutung haben für Sie die einzelnen Quellen, wenn Sie gesundheitsorientierte Informationen benötigen?**

| | | Überhaupt keine Bedeutung | | | | | Sehr hohe Bedeutung | | Keine Antwort |
|---|---|---|---|---|---|---|---|---|---|
| F6_1 | Familie | 1 | 2 | 3 | 4 | 5 | 6 | 7 | 8 |
| F6_2 | Freunde | 1 | 2 | 3 | 4 | 5 | 6 | 7 | 8 |
| F6_3 | Arzt/Ärztin | 1 | 2 | 3 | 4 | 5 | 6 | 7 | 8 |
| F6_4 | Apotheker/in | 1 | 2 | 3 | 4 | 5 | 6 | 7 | 8 |
| F6_5 | Versicherungsberater/in | 1 | 2 | 3 | 4 | 5 | 6 | 7 | 8 |
| F6_6 | Internet | 1 | 2 | 3 | 4 | 5 | 6 | 7 | 8 |
| F6_7 | Bücher/Medizinzeitschriften | 1 | 2 | 3 | 4 | 5 | 6 | 7 | 8 |
| F6_8 | Andere Quellen | 1 | 2 | 3 | 4 | 5 | 6 | 7 | 8 |

**How important are the following sources for you, if you need health related information?**

Adapted from [41,42]

| | | Not important at all | | | | | | Very important | No answer |
|---|---|---|---|---|---|---|---|---|---|
| F6_1 | Family | 1 | 2 | 3 | 4 | 5 | 6 | 7 | 8 |
| F6_2 | Friends | 1 | 2 | 3 | 4 | 5 | 6 | 7 | 8 |
| F6_3 | Physician | 1 | 2 | 3 | 4 | 5 | 6 | 7 | 8 |
| F6_4 | Pharmacist | 1 | 2 | 3 | 4 | 5 | 6 | 7 | 8 |
| F6_5 | Insurance agent | 1 | 2 | 3 | 4 | 5 | 6 | 7 | 8 |
| F6_6 | Internet | 1 | 2 | 3 | 4 | 5 | 6 | 7 | 8 |
| F6_7 | Books/journals | 1 | 2 | 3 | 4 | 5 | 6 | 7 | 8 |
| F6_8 | Other sources | 1 | 2 | 3 | 4 | 5 | 6 | 7 | 8 |

**Im Folgenden werden die Nutzung und die Einstellung hinsichtlich des Internets für die Suche nach gesundheitsorientierten Inhalten abgefragt.** *Unter dem Begriff* ***Internet*** *werden folgende Informationskanäle verstanden: Suchmaschinen, Wikis, Foren, Blogs, Social Networks, Elektronische Datenbanken und Apps. Zu* ***gesundheitsorientierten Inhalten/Informationen*** *zählen: Informationen über Krankheitszustände, Therapiemöglichkeiten, Medikamentenbeschreibungen, medizinische Forschungsergebnisse, Fitness, Wellness, Ernährung und Diäten.*

**6. Bitte kreuzen Sie an, wie häufig Sie die folgenden Kanäle im Internet für die Suche nach gesundheitsorientierten Informationen nutzen.**

| | | *Erläuterung* | Täglich | Wöchentlich | Seltener als wöchentlich | Monatlich | Seltener als monatlich | Nie |
|---|---|---|---|---|---|---|---|---|
| F7_1 | Suchmaschinen | *Durch die Eingabe von Schlagworten kann nach Dokumenten und Dateien im World Wide Web gesucht werden – Beispiele: Google, Bing, Yahoo!, www.medvista.de* | 1 | 2 | 3 | 4 | 5 | 6 |
| F7_2 | Wikis | *Online-Enzyklopädien – Beispiele: Wikipedia, www.pflegewiki.de* | 1 | 2 | 3 | 4 | 5 | 6 |
| F7_3 | Elektronische Datenbanken/Zeitschriften | *Zugriff auf wissenschaftliche Artikel und Studien– Beispiele: www.bmj.com, www.eMedicine.com, www.Pubmed.gov* | 1 | 2 | 3 | 4 | 5 | 6 |
| F7_4 | E-Mail | *Elektronische/r Post bzw. Brief* | 1 | 2 | 3 | 4 | 5 | 6 |
| F7_5 | Social Networks/Mikroblogging | *Netzwerke, in denen die Benutzer gemeinsam eigene Inhalte erstellen und über ihren aktuellen Status informieren können – Beispiele: Xing, Facebook, Friendster, MySpace, Twitter, tumblr, friendfeed* | 1 | 2 | 3 | 4 | 5 | 6 |

| | | | | | | | | |
|---|---|---|---|---|---|---|---|---|
| F7_6 | Gesundheitsforen/ -blogs | *Virtueller Raum, um sich zu bestimmten Themen auszutauschen bzw. die Möglichkeit ein Online-Tagebuch zu verfassen – Beispiele: imedo.de, frag-dich-gesund.de* | 1 | 2 | 3 | 4 | 5 | 6 |
| F7_7 | Podcasts | *Bereitstellung und Rezeption von kurzen Hör-/Videobeiträgen zu bestimmten Themen – Beispiele: YouTube, Viddler* | 1 | 2 | 3 | 4 | 5 | 6 |
| F7_8 | Videokonferenz | *Kommunikation erfolgt über den Computer mittels Kamera und Mikrofon – Beispiel: Skype* | 1 | 2 | 3 | 4 | 5 | 6 |
| F7_9 | Instant Messaging/Chat | *Zwei oder mehrere TeilnehmerInnen unterhalten sich mittels Textnachrichten – Beispiele: Skype, ICQ* | 1 | 2 | 3 | 4 | 5 | 6 |
| F7_10 | Apps (Applikationen) | *Eine Applikation (kurz: App) ist eine Anwendung auf Smartphones – vergleichbar mit einem Programm am Computer.* | 1 | 2 | 3 | 4 | 5 | 6 |

**The following section asks questions about the usage and attitude toward the Internet for health-related information searches.** *For the purposes of this questionnaire, the term* ***Internet*** *comprises the following information channels: search engines, wikis, fora, blogs, social networks, electronic databases and apps. The following information ranks among* ***health-related information****: Information about medical conditions, therapy options, prescriptions of drugs, medical research results, fitness, wellness, nutrition and diets.*

**Please indicate how often you use the following channels on the Internet for health-related information searches.**

| | | *Annotation* | daily | weekly | less often than weekly | monthly | less often than monthly | never |
|---|---|---|---|---|---|---|---|---|
| F7_1 | Search engines | *Through the entry of buzzwords, documents and data files can be searched on the WWW – examples: Google, Bing, Yahoo!, www.medvista.de* | 1 | 2 | 3 | 4 | 5 | 6 |
| F7_2 | Wikis | *Online-Encyclopedias – Examples: Wikipedia, www.pflegewiki.de* | 1 | 2 | 3 | 4 | 5 | 6 |
| F7_3 | Electronic databases/ journals | *Access to scientific papers and studies – examples: www.bmj.com, www.eMedicine.com, www.Pubmed.gov* | 1 | 2 | 3 | 4 | 5 | 6 |
| F7_4 | E-mail | *Electronic mail and letters* | 1 | 2 | 3 | 4 | 5 | 6 |
| F7_5 | Social Networks/Mikro-blogging | *Networks, in which users jointly generate shared contents and can inform others about their own current status – examples: Xing, Facebook, Friendster, MySpace, Twitter, tumblr, friendfeed* | 1 | 2 | 3 | 4 | 5 | 6 |
| F7_6 | Health fora/-blogs | *Virtual space in order to compare notes about specific topics or the possibility to write an online-diary – Examples: imedo.de, frag-dich-gesund.de* | 1 | 2 | 3 | 4 | 5 | 6 |
| F7_7 | Podcasts | *Provision and reception of short audio/video contributions to specific topics – Examples: YouTube, Viddler* | 1 | 2 | 3 | 4 | 5 | 6 |
| F7_8 | Videoconferences | *Communication through the computer via electronic camera and microphone - examples: Skype* | 1 | 2 | 3 | 4 | 5 | 6 |
| F7_9 | Instant Messaging/Chat | *Two or more participants chat through text messages – examples: Skype, ICQ* | 1 | 2 | 3 | 4 | 5 | 6 |
| F7_10 | Apps | *An application (briefly: app) is a task on smartphones – comparable to a software program on a computer.* | 1 | 2 | 3 | 4 | 5 | 6 |

Adapted from [43]

7. **Bitte kreuzen Sie an, aus welchen Gründen Sie das Internet für die Suche nach gesundheitsorientierten Informationen nutzen?**

| | **Ich nutze das Internet, weil …** | Trifft über-haupt nicht zu | | | | | | Trifft voll-kommen zu | Keine Antwort |
|---|---|---|---|---|---|---|---|---|---|
| F11_1 | … ich auf einfache Art und Weise Recherchen machen kann. | 1 | 2 | 3 | 4 | 5 | 6 | 7 | 8 |
| F11_2 | … es die Informationssuche für mich erleichtert. | 1 | 2 | 3 | 4 | 5 | 6 | 7 | 8 |
| F11_3 | … es meine Produktivität bei der Suche nach gesundheitsorientierten Informationen steigert. | 1 | 2 | 3 | 4 | 5 | 6 | 7 | 8 |
| F11_4 | … ich schnell gelernt habe damit umzugehen. | 1 | 2 | 3 | 4 | 5 | 6 | 7 | 8 |
| F11_5 | … es mir eine Vielzahl an Informationen bietet. | 1 | 2 | 3 | 4 | 5 | 6 | 7 | 8 |
| F11_6 | … die Informationen aktuell sind. | 1 | 2 | 3 | 4 | 5 | 6 | 7 | 8 |
| F11_7 | … die Informationen einfach zu verstehen sind. | 1 | 2 | 3 | 4 | 5 | 6 | 7 | 8 |
| F11_8 | … es mir richtige Informationen bietet. | 1 | 2 | 3 | 4 | 5 | 6 | 7 | 8 |

| | | | | | | | | | | |
|---|---|---|---|---|---|---|---|---|---|---|
| F11_9 | ... es mir unterschiedliche Formate, wie z.B. Social Networks, Podcasts oder Gesundheitsforen bietet. | 1 | 2 | 3 | 4 | 5 | 6 | 7 | 8 |
| F11_10 | … ich Zeit sparen möchte. | 1 | 2 | 3 | 4 | 5 | 6 | 7 | 8 |
| F11_11 | … ich auf einfache Art und Weise mit jemandem in Kontakt treten kann. | 1 | 2 | 3 | 4 | 5 | 6 | 7 | 8 |
| F11_12 | … ich am Puls der Zeit sein möchte. | 1 | 2 | 3 | 4 | 5 | 6 | 7 | 8 |
| F11_13 | … ich mich lieber anonym informieren möchte. | 1 | 2 | 3 | 4 | 5 | 6 | 7 | 8 |
| F11_14 | … eine Nutzung 24 Stunden 7 Tage möglich ist. | 1 | 2 | 3 | 4 | 5 | 6 | 7 | 8 |
| F11_15 | … ich mein Wissen mit anderen teilen kann. | 1 | 2 | 3 | 4 | 5 | 6 | 7 | 8 |
| F11_16 | … dadurch der Sucherfolg von Informationen gesteigert wird. | 1 | 2 | 3 | 4 | 5 | 6 | 7 | 8 |
| F11_17 | … es Spaß macht, es zu nutzen. | 1 | 2 | 3 | 4 | 5 | 6 | 7 | 8 |
| F11_18 | … ich finde, dass es unterhaltsam ist. | 1 | 2 | 3 | 4 | 5 | 6 | 7 | 8 |

**Why do you use the Internet for health-related information searches? Please mark all answers that apply.**

| | I use the Internet because … | Strongly disagree | | | | | | Strongly agree | No answer | Adapted from |
|---|---|---|---|---|---|---|---|---|---|---|
| F11_1 | … I can search online easily. | 1 | 2 | 3 | 4 | 5 | 6 | 7 | 8 | [7,47,48] |
| F11_2 | … it simplifies the information search for me. | 1 | 2 | 3 | 4 | 5 | 6 | 7 | 8 | [9,21,44,63] |
| F11_3 | … it enhances my productivity in health-related information searching. | 1 | 2 | 3 | 4 | 5 | 6 | 7 | 8 | [9,21,44,63] |
| F11_4 | … I have learnt quickly how to handle it. | 1 | 2 | 3 | 4 | 5 | 6 | 7 | 8 | [9,44,63] |
| F11_5 | … it offers a variety of information. | 1 | 2 | 3 | 4 | 5 | 6 | 7 | 8 | [7,47] |
| F11_6 | … information is up-to-date. | 1 | 2 | 3 | 4 | 5 | 6 | 7 | 8 | [7,49] |
| F11_7 | … information can be understood easily. | 1 | 2 | 3 | 4 | 5 | 6 | 7 | 8 | [7,47] |
| F11_8 | … it offers the right information. | 1 | 2 | 3 | 4 | 5 | 6 | 7 | 8 | [44,63] |
| F11_9 | ... it offers different formats, like e.g. social networks, podcasts or health fora. | 1 | 2 | 3 | 4 | 5 | 6 | 7 | 8 | [7,47,50] |
| F11_10 | … I want to save time. | 1 | 2 | 3 | 4 | 5 | 6 | 7 | 8 | [7,48] |
| F11_11 | … I can establish contact with someone easily. | 1 | 2 | 3 | 4 | 5 | 6 | 7 | 8 | [7,49] |
| F11_12 | … I want to be up-to-date. | 1 | 2 | 3 | 4 | 5 | 6 | 7 | 8 | [7] |
| F11_13 | … I prefer to gather information anonymously. | 1 | 2 | 3 | 4 | 5 | 6 | 7 | 8 | [7,50] |
| F11_14 | … usage is possible for 24 hours on 7 days. | 1 | 2 | 3 | 4 | 5 | 6 | 7 | 8 | [7,50] |
| F11_15 | … I can share my knowhow with others. | 1 | 2 | 3 | 4 | 5 | 6 | 7 | 8 | [7] |
| F11_16 | … the success of finding information can be increased. | 1 | 2 | 3 | 4 | 5 | 6 | 7 | 8 | [9,44,63] |
| F11_17 | … it is fun to use. | 1 | 2 | 3 | 4 | 5 | 6 | 7 | 8 | [9,45,51] |
| F11_18 | … I find it entertaining. | 1 | 2 | 3 | 4 | 5 | 6 | 7 | 8 | [9,45,46] |

**8. Bitte geben Sie an, inwiefern Sie folgenden Statements zustimmen.**

| | | Trifft überhaupt nicht zu | | | | | | Trifft vollkommen zu | Keine Antwort |
|---|---|---|---|---|---|---|---|---|---|
| F12_1 | Vorausgesetzt ich habe Internetzugang, dann nutze ich es auch zur Suche für gesundheitsorientierte Informationen. | 1 | 2 | 3 | 4 | 5 | 6 | 7 | 8 |
| F12_2 | Ich bin froh, wenn ich neue Suchmöglichkeiten im Internet für gesundheitsorientierte Informationen kennenlerne. | 1 | 2 | 3 | 4 | 5 | 6 | 7 | 8 |
| F12_3 | Ich finde im Internet immer die Informationen, die ich brauche. | 1 | 2 | 3 | 4 | 5 | 6 | 7 | 8 |
| F12_4 | Das Internet bietet die Möglichkeit, sich mit nationalen und internationalen Experten des Gesundheitswesens auszutauschen. | 1 | 2 | 3 | 4 | 5 | 6 | 7 | 8 |
| F12_5 | Ich betrachte das Web 2.0 als wichtige Informationsquelle für mein Gesundheitsinteresse. | 1 | 2 | 3 | 4 | 5 | 6 | 7 | 8 |
| F12_6 | Ich betrachte das Web 2.0 als relevant für die Suche nach gesundheitsorientierten Informationen. | 1 | 2 | 3 | 4 | 5 | 6 | 7 | 8 |
| F12_7 | Personen, die mir wichtig sind, schlagen mir für die Suche nach gesundheitsorientierten Inhalten das Web 2.0 vor. | 1 | 2 | 3 | 4 | 5 | 6 | 7 | 8 |
| F12_8 | Personen, zu denen ich aufsehe, schlagen mir für die Suche nach gesundheitsorientierten Inhalten das Web 2.0 vor. | 1 | 2 | 3 | 4 | 5 | 6 | 7 | 8 |

**Please indicate the extent to which the following statements apply to you.**

| | | Strongly disagree | | | | | | strongly agree | No answer | Adapted from |
|---|---|---|---|---|---|---|---|---|---|---|
| F12_1 | Assuming that I have access to the Internet, then I also use it for health information searches. | 1 | 2 | 3 | 4 | 5 | 6 | 7 | 8 | [18,64] |
| F12_2 | I am glad whenever I get to know new possibilities of health information searching on the Internet. | 1 | 2 | 3 | 4 | 5 | 6 | 7 | 8 | [65] |
| F12_3 | On the Internet I always find the kind of information I need. | 1 | 2 | 3 | 4 | 5 | 6 | 7 | 8 | [65] |
| F12_4 | The Internet offers the chance to compare notes with national and international experts from the health sector. | 1 | 2 | 3 | 4 | 5 | 6 | 7 | 8 | Edited by the research team |
| F12_5 | I consider Web 2.0 to be an important source of information for my health interests. | 1 | 2 | 3 | 4 | 5 | 6 | 7 | 8 | |
| F12_6 | I consider Web 2.0 to be a relevant source of health-related information. | 1 | 2 | 3 | 4 | 5 | 6 | 7 | 8 | |
| F12_7 | Individuals, who are important to me, suggest that I use Web 2.0 for health information searches. | 1 | 2 | 3 | 4 | 5 | 6 | 7 | 8 | Adapted from [9,11,17,62,63,66,67] |
| F12_8 | Individuals, who I look up to, suggest that I use Web 2.0 for health information searches. | 1 | 2 | 3 | 4 | 5 | 6 | 7 | 8 | [9,11,17,62,63,66,67] |

## INTERNETKOMMUNIKATION MIT DEM ARZT / INTERNET COMMUNICATION WITH THE PHYSICIAN

**9. Im Folgenden werden Fragen zum Thema Internetkommunikation mit Ihrem Allgemeinarzt/Ihrer Allgemeinärztin behandelt. Bitte kreuzen Sie an, wie häufig Sie das Internet für die Kommunikation mit Ihrem Allgemeinarzt/Ihrer Allgemeinärztin nutzen.**

| | Wie häufig nutzen Sie das Internet, um … | Täglich | Wöchentlich | Seltener als wöchentlich | Monatlich | Seltener als monatlich | Nie |
|---|---|---|---|---|---|---|---|
| F13 | … mit Ihrer/m Allgemeinärztin/Allgemeinarzt zu kommunizieren? | 1 | 2 | 3 | 4 | 5 | 6 |

**The following question relates to the topic of Internet communication with your general practitioner (GP). Please indicate how often you use the Internet for the communication with your GP at present.**

Edited by the research team

| | How often do you use the Internet to … | daily | weekly | less often than weekly | monthly | less often than monthly | never |
|---|---|---|---|---|---|---|---|
| F13 | … communicate with your general practitioner (GP)? | 1 | 2 | 3 | 4 | 5 | 6 |

**10. Können Sie sich vorstellen, in der Zukunft, häufiger mit Ihrem Allgemeinarzt/Ihrer Allgemeinärztin über das Internet zu kommunizieren?**

| Kann ich mir überhaupt nicht vorstellen | | | | | | Kann ich mir sehr gut vorstellen | Keine Antwort |
|---|---|---|---|---|---|---|---|
| 1 | 2 | 3 | 4 | 5 | 6 | 7 | 8 |

**Can you imagine using the Internet more often in the future for communicating with your GP?**

Edited by the research team

| | Highly unlikely | | | | | Very likely | No answer |
|---|---|---|---|---|---|---|---|
| F15_1 | 1 | 2 | 3 | 4 | 5 | 6 | 7 | 8 |

11. **Für welche der folgenden Bereiche könnten Sie sich vorstellen, dass die persönliche Kommunikation mit Ihrem Allgemeinarzt/Ihrer Allgemeinärztin durch die Internetkommunikation ersetzt wird?**

| | | Überhaupt nicht vorstellbar | | | | | | Sehr gut vorstellbar | Keine Antwort |
|---|---|---|---|---|---|---|---|---|---|
| F17_1 | Terminvereinbarungen | 1 | 2 | 3 | 4 | 5 | 6 | 7 | 8 |
| F17_2 | Erstauskünfte | 1 | 2 | 3 | 4 | 5 | 6 | 7 | 8 |
| F17_3 | Verschreibung von Rezepten | 1 | 2 | 3 | 4 | 5 | 6 | 7 | 8 |
| F17_4 | Krankmeldungen/Gesundschreiben | 1 | 2 | 3 | 4 | 5 | 6 | 7 | 8 |
| F17_5 | Überweisungen zu anderen ÄrztInnen | 1 | 2 | 3 | 4 | 5 | 6 | 7 | 8 |
| F17_6 | Besprechung von „normalen“ Testergebnissen | 1 | 2 | 3 | 4 | 5 | 6 | 7 | 8 |
| F17_7 | Besprechung von „kritischen“ Testergebnissen | 1 | 2 | 3 | 4 | 5 | 6 | 7 | 8 |
| F17_8 | Nachkontrollen | 1 | 2 | 3 | 4 | 5 | 6 | 7 | 8 |
| F17_9 | Betreuung von chronisch Kranken | 1 | 2 | 3 | 4 | 5 | 6 | 7 | 8 |
| F17_10 | Nebenwirkungen von Medikamenten | 1 | 2 | 3 | 4 | 5 | 6 | 7 | 8 |
| F17_11 | Routinebehandlungen (Halsschmerzen, Schnupfen etc.) | 1 | 2 | 3 | 4 | 5 | 6 | 7 | 8 |
| F17_12 | Psychotherapie | 1 | 2 | 3 | 4 | 5 | 6 | 7 | 8 |
| F17_13 | Mentale Gesundheitsprobleme | 1 | 2 | 3 | 4 | 5 | 6 | 7 | 8 |
| F17_14 | Akute Beschwerde (z.B. Brustschmerzen) | 1 | 2 | 3 | 4 | 5 | 6 | 7 | 8 |

**For which of the following areas could you imagine the replacement of personal communication with your general practitioner (GP) through Internet communication in the future?**

| | | highly unlikely | | | | | | very likely | No answer |
|---|---|---|---|---|---|---|---|---|---|
| F17_1 | Fixing of personal appointments | 1 | 2 | 3 | 4 | 5 | 6 | 7 | 8 |
| F17_2 | Preliminary advice | 1 | 2 | 3 | 4 | 5 | 6 | 7 | 8 |
| F17_3 | Writing of prescriptions | 1 | 2 | 3 | 4 | 5 | 6 | 7 | 8 |
| F17_4 | Doctor’s notes/certificates of health | 1 | 2 | 3 | 4 | 5 | 6 | 7 | 8 |
| F17_5 | Referrals to other doctors | 1 | 2 | 3 | 4 | 5 | 6 | 7 | 8 |
| F17_6 | Discussion of “normal” test results | 1 | 2 | 3 | 4 | 5 | 6 | 7 | 8 |
| F17_7 | Discussion of “critical” test results | 1 | 2 | 3 | 4 | 5 | 6 | 7 | 8 |
| F17_8 | Follow-up checks after treatment | 1 | 2 | 3 | 4 | 5 | 6 | 7 | 8 |
| F17_9 | Supervision of chronically ill people | 1 | 2 | 3 | 4 | 5 | 6 | 7 | 8 |
| F17_10 | Secondary effects of drugs | 1 | 2 | 3 | 4 | 5 | 6 | 7 | 8 |
| F17_11 | Routine treatments (sore throat, head cold etc.) | 1 | 2 | 3 | 4 | 5 | 6 | 7 | 8 |
| F17_12 | Psychotherapy | 1 | 2 | 3 | 4 | 5 | 6 | 7 | 8 |
| F17_13 | Mental health problems | 1 | 2 | 3 | 4 | 5 | 6 | 7 | 8 |
| F17_14 | Acute disorders (e.g. chest pains) | 1 | 2 | 3 | 4 | 5 | 6 | 7 | 8 |

Edited by the research team

12. **Wie wichtig ist es Ihnen, auch eine Online-Behandlung nutzen zu können?**

| | Ist mir überhaupt nicht wichtig. | | | | | | Ist mir sehr wichtig. | Keine Antwort |
|---|---|---|---|---|---|---|---|---|
| F18_1 | 1 | 2 | 3 | 4 | 5 | 6 | 7 | 8 |

**How important is it to you to be able to use online-treatment as well?**

| | Not important at all | | | | | | Very important | No answer |
|---|---|---|---|---|---|---|---|---|
| F18_1 | 1 | 2 | 3 | 4 | 5 | 6 | 7 | 8 |

Edited by the research team

13. **Inwiefern wären Sie bereit für eine Online-Behandlung einen gewissen Betrag zuzuzahlen?**

| | Ich wäre dazu überhaupt nicht bereit. | | | | | | Ich wäre dazu bereit. | Keine Antwort |
|---|---|---|---|---|---|---|---|---|
| F19_1 | 1 | 2 | 3 | 4 | 5 | 6 | 7 | 8 |

**Indicate how willing you would be to pay a certain amount additionally for online-treatment?**

| | I would not be willing at all | | | | | | I would be willing | No answer |
|---|---|---|---|---|---|---|---|---|
| F19_1 | 1 | 2 | 3 | 4 | 5 | 6 | 7 | 8 |

Edited by the research team

**Bitte geben Sie im Folgenden an, wie sehr die einzelnen Aussagen auf Sie zutreffen.**

| | | Trifft über-haupt nicht zu | | | | | | Trifft vollkommen zu | Keine Antwort |
|---|---|---|---|---|---|---|---|---|---|
| F20_1 | Es ist wichtig, einem Arzt bzw. einer Ärztin bereits gut informiert gegenüberzutreten. | 1 | 2 | 3 | 4 | 5 | 6 | 7 | 8 |
| F20_2 | Wenn ich mich über Krankheiten im Internet informiere, habe ich das Bedürfnis, mit meinem Arzt bzw. meiner Ärztin darüber zu sprechen. | 1 | 2 | 3 | 4 | 5 | 6 | 7 | 8 |
| F20_3 | Wenn ich eine Therapie verschrieben bekomme, erkundige ich mich nach Alternativtherapien im Internet. | 1 | 2 | 3 | 4 | 5 | 6 | 7 | 8 |
| F20_4 | Manchmal habe ich das Gefühl, besser über meinen Zustand informiert zu sein, als mein Arzt bzw. meine Ärztin. | 1 | 2 | 3 | 4 | 5 | 6 | 7 | 8 |
| F20_5 | Ist der Patient/die Patientin informiert, wird die Kommunikation mit dem Arzt/der Ärztin dadurch verbessert. | 1 | 2 | 3 | 4 | 5 | 6 | 7 | 8 |
| F20_6 | Erst nach einer Recherche im Internet entscheide ich, ob ein Arztbesuch notwendig ist. | 1 | 2 | 3 | 4 | 5 | 6 | 7 | 8 |
| F20_7 | Wenn mir Medikamente verschrieben wurden, suche ich Informationen darüber im Internet. | 1 | 2 | 3 | 4 | 5 | 6 | 7 | 8 |
| F20_8 | Ist der Patient/die Patientin informiert, nimmt sich der Arzt/die Ärztin mehr Zeit für die Behandlung. | 1 | 2 | 3 | 4 | 5 | 6 | 7 | 8 |
| F20_9 | Der Arzt/die Ärztin verschreibt eher ein gewünschtes Medikament, wenn der Patient/die Patientin informiert ist. | 1 | 2 | 3 | 4 | 5 | 6 | 7 | 8 |

**Please indicate how well the following statements apply to you.**

| | | Strongly disagree | | | | | | Strongly agree | No answer | |
|---|---|---|---|---|---|---|---|---|---|---|
| F20_1 | It is important to me to be well-informed when consulting a physician. | 1 | 2 | 3 | 4 | 5 | 6 | 7 | 8 | Adapted from [15,55] |
| F20_2 | When I obtain health information from the Internet, I need to talk about this information with my physician. | 1 | 2 | 3 | 4 | 5 | 6 | 7 | 8 | Edited by the research team |
| F20_3 | When a therapy is prescribed for me, I look for alternative therapies on the Internet. | 1 | 2 | 3 | 4 | 5 | 6 | 7 | 8 | Adapted from [55,56] |
| F20_4 | Sometimes I have the feeling that I am better informed about my medical condition than my physician. | 1 | 2 | 3 | 4 | 5 | 6 | 7 | 8 | Edited by the research team |
| F20_5 | If the patient is informed, the communication with the physician is improved. | 1 | 2 | 3 | 4 | 5 | 6 | 7 | 8 | |
| F20_6 | I only decide whether a consultation with a physician is really necessary, once I have conducted some health information searches on the Internet. | 1 | 2 | 3 | 4 | 5 | 6 | 7 | 8 | |
| F20_7 | If some medicines have been prescribed, I look for information about them on the Internet. | 1 | 2 | 3 | 4 | 5 | 6 | 7 | 8 | Adapted from [55, 56] |
| F20_8 | If the patient is informed, the physician allows more time for the treatment. | 1 | 2 | 3 | 4 | 5 | 6 | 7 | 8 | Edited by the research team |
| F20_9 | The physician is more likely to prescribe a requested medicine, if the patient is informed. | 1 | 2 | 3 | 4 | 5 | 6 | 7 | 8 | |

## EINSTELLUNG ZUM ARZT/ARZTDATEN/ARZTKRITERIEN SOLL-IST-ZUSTAND/ATTITUDE TOWARDS THE PHYSICIAN/PHYSICIAN DATA/PHYSICIAN DATA TARGET-PERFORMANCE

**14. Wie gut sind Sie über Ihren Krankheitszustand informiert, wenn Sie zu Ihrem Allgemeinarzt/Ihrer Allgemeinärztin gehen?**

| Überhaupt nicht gut | | | | | | Sehr gut | Keine Antwort |
|---|---|---|---|---|---|---|---|
| 1 | 2 | 3 | 4 | 5 | 6 | 7 | 8 |

**How well informed are you about your medical state when you consult a general practitioner?**

Edited by the research team

| Not well at all | | | | | | Very well | No answer |
|---|---|---|---|---|---|---|---|
| 1 | 2 | 3 | 4 | 5 | 6 | 7 | 8 |

## GESUNDHEIT UND ARZTBEZIEHUNG / HEALTH AND RELATIONSHIP WITH THE PRACTITIONER

**15. Bitte beantworten Sie nachfolgende Fragen zur Ihrem Gesundheits- und Ernährungsbewusstsein. Kreuzen Sie an, wie sehr die angeführten Statements auf Sie zutreffen.**

| | | Trifft überhaupt nicht zu | | | | | | Trifft vollkommen zu | Keine Antwort |
|---|---|---|---|---|---|---|---|---|---|
| F42_1 | Meine Gesundheit ist mir wichtig. | 1 | 2 | 3 | 4 | 5 | 6 | 7 | 8 |
| F42_2 | Ich achte auf meine Ernährung. | 1 | 2 | 3 | 4 | 5 | 6 | 7 | 8 |
| F42_3 | Ich fühle mich körperlich fit. | 1 | 2 | 3 | 4 | 5 | 6 | 7 | 8 |
| F42_4 | Ich treibe regelmäßig Sport. | 1 | 2 | 3 | 4 | 5 | 6 | 7 | 8 |
| F42_5 | Ich gehe ungern zum Arzt. | 1 | 2 | 3 | 4 | 5 | 6 | 7 | 8 |
| F42_6 | Wenn möglich vermeide ich die Einnahme von Medizin. | 1 | 2 | 3 | 4 | 5 | 6 | 7 | 8 |
| F42_7 | Ich bevorzuge die Einnahme von homöopathischen Mitteln. | 1 | 2 | 3 | 4 | 5 | 6 | 7 | 8 |
| F42_8 | Ich nehme vorwiegend verschreibungspflichtige Medikamente. | 1 | 2 | 3 | 4 | 5 | 6 | 7 | 8 |
| F42_9 | Ich gehe regelmäßig zu Vorsorgeuntersuchungen. | 1 | 2 | 3 | 4 | 5 | 6 | 7 | 8 |

**Please answer the following questions about your health and nutrition awareness. Please indicate how well the following statements apply to you.**

| | | Strongly disagree | | | | | | Strongly agree | No answer |
|---|---|---|---|---|---|---|---|---|---|
| F42_1 | My health is very important to me. | 1 | 2 | 3 | 4 | 5 | 6 | 7 | 8 |
| F42_2 | I am careful about what I eat. | 1 | 2 | 3 | 4 | 5 | 6 | 7 | 8 |
| F42_3 | I feel physically fit. | 1 | 2 | 3 | 4 | 5 | 6 | 7 | 8 |
| F42_4 | I practice sports on a regular basis. | 1 | 2 | 3 | 4 | 5 | 6 | 7 | 8 |
| F42_5 | I am reluctant to visit a physician. | 1 | 2 | 3 | 4 | 5 | 6 | 7 | 8 |
| F42_6 | Whenever possible I avoid taking medicine. | 1 | 2 | 3 | 4 | 5 | 6 | 7 | 8 |
| F42_7 | I prefer to take homeopathic remedies. | 1 | 2 | 3 | 4 | 5 | 6 | 7 | 8 |
| F42_8 | Predominantly I take remedies, which are only available on prescription. | 1 | 2 | 3 | 4 | 5 | 6 | 7 | 8 |
| F42_9 | I have regular preventive medical examinations. | 1 | 2 | 3 | 4 | 5 | 6 | 7 | 8 |

Adapted from [15,60] [15] [61] [15,61]

Edited by the research team

Adapted from [41]

## DEMOGRAPHISCHE ANGABEN / SOCIODEMOGRAPHIC DATA

**D1 Geschlecht:** [1] männlich
[2] weiblich

**D2_1 Geburtsjahr:** ____________

**D1 Gender:** [1] male
[2] female

**D2_1 Year of birth:** ____________

**Höchste abgeschlossene Ausbildung:**

1 Schüler in allgemeinbildender Schule (ohne Schulabschluss)
2 Haupt-/Volksschulabschluss ohne Lehre
3 Haupt-/Volksschulabschluss mit Lehre
4 Weiterführende Schule ohne Abitur (Realschulabschluss / Mittlere Reife)
5 Abitur / (Fach-) Hochschulreife ohne Studium
6 Abitur / (Fach-) Hochschulreife mit Studium
7 Promotion/ Habilitation
8 Keine Antwort

**Highest level of education:** (D4)

1 Without school qualification
2 Secondary general school
3 Polytechnic secondary school
4 Intermediate secondary school
5 High school diploma / A-levels
6 University degree
7 Postdoctoral degree / Professor
8 No answer

**Familienstand:**

1 Ledig
2 In einer Partnerschaft
3 Verheiratet
4 Geschieden
5 Verwitwet
6 Keine Antwort

**Marital status:** (D5)

1 Single
2 Close-partnered
3 Married
4 Divorced
5 Widowed
6 No answer

D6_1 **Anzahl der Personen im Haushalt:** ___________

D6_1 **Number of individuals in the household:** ___________

**Monatliches Nettohaushaltseinkommen:**

1 < 1.500 € 2 1.500–2.500 3 2.501–3.500 4 3.501–4.500 5 > 4.500 6 Keine Antwort

**Monthly household net income:** (D8)

1 < 1.500 € 2 1.500–2.500 3 2.501–3.500 4 3.501–4.500 5 > 4.500 6 No answer
