# Supplementary material for: Gender Differences in Searching for Health Information on the Internet and the Virtual Patient-Physician Relationship in Germany: Exploratory Results on How Men and Women Differ and Why
Source: J Med Internet Res. 2015 Jun 22;17(6):e156. doi: 10.2196/jmir.4127 (PMC4526954; doi:10.2196/jmir.4127)
Supplement: Multimedia Appendix 2 [file jmir_v17i6e156_app2.pdf]

Table A

Rotated Factor Component Matrix for the Exploratory Factor Analysis of Motivational Variables Influencing Internet Health Information Searching (EFA 1)

Factor 1: Social motive and joyousness of Internet health information searching

Factor 2: Perceived usefulness of the Internet for health information searching

Factor 3: Usefulness of the information gained from Internet health information searching

**Variable name:** **Original item wording (German)** (Antwortskala: 1= trifft überhaupt nicht zu, 7=trifft vollkommen zu, 8=keine Antwort)	**Variable name:** **Item translation (English)** (answer scale: 1=strongly disagree, 7=strongly agree, 8=no answer)	**Component**		
		**1**	**2**	**3**
**Ich nutze das Internet, weil …**	**I use the Internet because …**			
F11_15 ... ich mein Wissen mit anderen teilen kann.	F11_15 … I can share my knowhow with others.	**.816**	.052	.123
F11_12 ... ich am Puls der Zeit sein möchte.	F11_12 … I want to be up-to-date.	**.793**	.044	.280
F11_11 ... ich auf einfache Art und Weise mit jemandem in Kontakt treten kann.	F11_11 … I can establish contact with someone easily.	**.788**	.128	.164
F11_9 ... es mir unterschiedliche Formate, wie z.B. Social Networks, Podcasts oder Gesundheitsforen bietet.	F11_9 ... it offers different formats, like e.g. social networks, podcasts or health fora.	**.736**	.059	.201
F11_18 ... ich finde, dass es unterhaltsam ist.	F11_18 … I find it entertaining.	**.673**	.255	.193
F11_13 ... ich mich lieber anonym informieren möchte.	F11_13 … I prefer to gather information anonymously.	**.638**	.231	.028
F11_16 ... dadurch der Sucherfolg von Informationen gesteigert wird.	F11_16 … the success of finding information can be increased.	**.590**	.360	.252
F11_2 ... es die Informationssuche für mich erleichtert.	F11_2 … it simplifies the information search for me.	.073	**.864**	.251
F11_1 ... ich auf einfache Art und Weise Recherchen machen kann.	F11_1 … I can search online easily.	.052	**.855**	.132
F11_5 ... es mir eine Vielzahl an Informationen bietet.	F11_5 … it offers a variety of information	.119	**.760**	.420

Variable name: Original item wording (German)	Variable name: Item translation (English)	Component		
		**1**	**2**	**3**
**Ich nutze das Internet, weil …**	**I use the Internet because …**			
**F11_14** ... eine Nutzung 24 Stunden 7 Tage möglich ist.	**F11_14** … usage is possible for 24 hours on 7 days.	.252	**.720**	,.18
**F11_4** ... ich schnell gelernt habe damit umzugehen.	**F11_4** … I have learnt quickly how to handle it.	.203	**.700**	.364
**F11_3** ... es meine Produktivität bei der Suche nach gesundheitsorientierten Informationen steigert.	**F11_3** … it enhances my productivity for health-related information searching.	.287	**.600**	.313
**F11_7** ... die Informationen einfach zu verstehen sind.	**F11_7** … information can be understood easily.	.262	.324	**.789**
**F11_8** ... es mir richtigen Informationen bietet.	**F11_8** … it offers the right information.	.347	.220	**.778**
**F11_6** ... die Informationen aktuell sind.	**F11_6** … information is up-to-date.	.221	.379	**.767**

Table A (Continuation)

Methodological details of the results of EFA 1

An EFA of the 18 items measuring the underlying motives for Internet health information searching lead to a three-factor solution explaining 66.69 % of variance for the purified scale (number of factors according to the eigenvalue-criterion and the scree-test, extraction method: principal component analysis, rotation method: varimax with Kaiser-normalization, rotation converged in 5 iterations). As required, the Kaiser-Meyer-Olkin-measure of the appropriateness of the sample (KMO-Test) was non significant (P=.93), the Bartlett-Test of sphericity was significant (Chi-square$_{120}$=8345.2, P<.001). Two items were excluded from further analysis due to weak factor loadings. The reduced scale of 16 items lead to a three-factor solution for the motivational variables underlying Internet health information searching. The first factor (eigenvalue=7.28) consisted of 7 items featuring the social motive and joyousness of Internet health information searching, the second factor (eigenvalue=2.38) comprised 6 items representing perceived usefulness of the Internet as a medium for health information searching and, last but not least, the third factor (eigenvalue=1.01) was construed by 3 items focusing on the usefulness of the information gained from the Internet for health information searching. Table A shows the full rotated factor component matrix.

Table B

Rotated Factor Component Matrix for the Exploratory Factor Analysis of Attitudinal Variables Influencing Internet Health Information Searching (EFA 2)

Factor 1: Health and nutrition awareness

Factor 2: Reluctance to make use of medical support

**Variable name: Original item wording (German)** (Antwortskala: 1= trifft überhaupt nicht zu, 7=trifft vollkommen zu, 8=keine Antwort)	**Variable name: Item translation (English)** (answer scale: 1=strongly disagree, 7=strongly agree, 8=no answer)	**Component**	
		**1**	**2**
F42_2 Ich achte auf meine Ernährung.	F42_2 I am careful about what I eat.	**.796**	-.009
F42_3 Ich fühle mich körperlich fit.	F42_3 I feel physically fit.	**.787**	.185
F42_4 Ich treibe regelmäßig Sport.	F42_4 I practice sports on a regular basis.	**.729**	.190
F42_1 Meine Gesundheit ist mir wichtig.	F42_1 My health is very important to me.	**.668**	-.095
F42_5 Ich gehe ungern zum Arzt.	F42_5 I am reluctant to visit a physician.	-.108	**.829**
F42_6 Wenn möglich vermeide ich die Einnahme von Medizin.	F42_6 Whenever possible I avoid taking medicine.	.247	**.774**

Methodological details of the results of EFA 2

An EFA of the 9 items measuring the attitudinal influences deriving from different health and nutrition awareness and proneness to use medical support lead to a two-factor solution explaining 61.14 % of variance for the purified scale (number of factors according to the eigenvalue-criterion, principal component analysis, varimax-rotation with Kaiser-normalization, rotation converged in 3 iterations). As required, the Kaiser-Meyer-Olkin-measure of the appropriateness of the sample (KMO-Test) was non significant (P=.69), the Bartlett-Test of sphericity was significant (Chi-square$_{15}$=1168.3, P<.001). Three items were eliminated from further calculation because of low factor loadings or loadings on more than one factor with a factor loading above .45.

The scale with the 6 remaining items lead to a two-factor solution for the attitudinal variables underlying Internet health information searching. The first factor (eigenvalue=2.40) consisted of 4 items featuring the nutrition and health awareness on Internet health information searching, the second factor (eigenvalue=1.27) comprised 2 items representing the reluctance to make use of medical support. Table B shows the full rotated factor component matrix.

Table C

Factor Component Matrix for the Exploratory Factor Analysis of the Personal Disposition of Being Well-informed as a Patient (EFA 3)

**Variable name:** **Original item wording (German)** (Antwortskala: 1= trifft überhaupt nicht zu, 7=trifft vollkommen zu, 8=keine Antwort)	**Variable name:** **Item translation (English)** (answer scale: 1=strongly disagree, 7=strongly agree, 8=no answer)	**Component** **1**
F20_8: Ist der Patient/die Patientin informiert, nimmt sich der Arzt/die Ärztin mehr Zeit für die Behandlung.	F20_8: If the patient is informed, the physician allows more time for the treatment.	**.805**
F20_9: Der Arzt/die Ärztin verschreibt eher ein gewünschtes Medikament, wenn der Patient/die Patientin informiert ist.	F20_9: The physician is more likely to prescribe a requested medicine, if the patient is informed.	**.788**
F20_3: Wenn ich eine Therapie verschrieben bekomme, erkundige ich mich nach Alternativtherapien im Internet.	F20_3: When a therapy is prescribed for me, I look for alternative therapies on the Internet.	**.771**
F20_5: Ist der Patient/die Patientin informiert, wird die Kommunikation mit dem Arzt/der Ärztin dadurch verbessert.	F20_5: If the patient is informed, the communication with the physician is improved.	**.737**
F20_4: Manchmal habe ich das Gefühl, besser über meinen Zustand informiert zu sein, als mein Arzt bzw. meine Ärztin.	F20_4: Sometimes I have the feeling that I am better informed about my medical condition than my physician.	**.718**
F20_7: Wenn mir Medikamente verschrieben wurden, suche ich Informationen darüber im Internet.	F20_7: If some medicines have been prescribed, I look for information about them on the Internet.	**.700**
F20_1: Es ist wichtig, einem Arzt bzw. einer Ärztin bereits gut informiert gegenüberzutreten. (	F20_1: It is important to me to be well-informed when consulting a physician.	**.694**
F20_6: Erst nach einer Recherche im Internet entscheide ich, ob ein Arztbesuch notwendig ist.	F20_6: I only decide whether a consultation with a physician is really necessary, once I have conducted some health information searches on the Internet.	**.689**
F20_2: Wenn ich mich über Krankheiten im Internet informiere, habe ich das Bedürfnis, mit meinem Arzt bzw. meiner Ärztin darüber zu sprechen.	F20_2: When I obtain health information from the Internet, I need to talk about this information with my physician.	**.627**

Methodological details of the results of EFA 3

An EFA of the 9 items measuring the personal disposition of being well-informed as a patient lead to a single factor solution explaining 52.93 % of variance (number of factors according to the eigenvalue-criterion and the scree-test, principal component analysis without necessity of rotation) with an eigenvalue of 4.76. As required, the Kaiser-Meyer-Olkin-measure of the appropriateness of the sample (KMO-Test) was non significant (P=.90), the Bartlett-Test of sphericity was significant (Chi-$square_{36}$=3643.3, P<.001). All items were used for further calculation. Table C shows the full factor component matrix.

Table D

Rotated Factor Component Matrix for the Exploratory Factor Analysis of Variables Measuring Situational and Normative Influences on Internet Health Information Searching (EFA 4)

Factor 1: Situational influences on Internet health information searching

Factor 2: Normative influences on Internet health information searching

**Variablenname:** **Original item wording (German)** (Antwortskala: 1=trifft überhaupt nicht zu, 7=trifft vollkommen zu, 8=keine Antwort)	**Variable name:** **Item translation (English)** (answer scale: 1=strongly disagree, 7=strongly agree, 8=no answer)	**Component**	
		**1**	**2**
F12_1 Vorausgesetzt ich habe Internetzugang, dann nutze ich es auch zur Suche für gesundheitsorientierte Informationen.	F12_1 Assuming that I have access to the Internet, then I also use it for health information searches.	**.912**	-.002
F12_2 Ich bin froh, wenn ich neue Suchmöglichkeiten im Internet für gesundheitsorientierte Informationen kennenlerne.	F12_2 I am glad whenever I get to know new possibilites of health information searching on the Internet.	**.857**	.239
F12_3 Ich finde im Internet immer die Informationen, die ich brauche.	F12_3 On the Internet I always find the kind of information I need.	**.613**	.274
F12_8 Personen, zu denen ich aufsehe, schlagen mir für die Suche nach gesundheitsorientierten Inhalten das Web 2.0 vor.	F12_8 Individuals, who I look up to, suggest that I use Web 2.0 for health information searches.	.155	**.956**
F12_7 Personen, die mir wichtig sind, schlagen mir für die Suche nach gesundheitsorientierten Inhalten das Web 2.0 vor.	F12_7 Individuals, who are important to me, suggest that I use Web 2.0 for health information searches.	.201	**.944**

Methodological details of the results of EFA 4

An EFA of the 5 items measuring the underlying situational and normative influences on Internet health information searching lead to a two-factor solution explaining 78.88 % of variance (number of factors according to the eigenvalue-criterion and the scree-test, principal component analysis, varimax-rotation with Kaiser-normalization, rotation converged in 3 iterations). As required, the Kaiser-Meyer-Olkin-measure of the appropriateness of the sample (KMO-Test) was non significant (*P*=.63), the Bartlett-Test of sphericity was significant (Chi-square_{10}=2250.6, *P*<.001). All items were used for further calculation. The scale lead to a two-factor solution for the situational and involvement variables underlying Internet health information searching. The first factor (eigenvalue=2.68) consisted of 3 items featuring the situational influences on Internet health information searching, the second factor (eigenvalue=1.26) comprised 2 items representing the normative influences. Table D shows the full rotated factor component matrix.

Table E

Formulas for Calculating Weighted Means of Factor Sum Scores Used in the *t*-Tests: SPSS-Syntax (Tables 3 – 6 of the paper)

Table 3

Factor name	Formula (SPSS-Syntax)
Factor 1: Social motive and joyousness of Internet health information searching	COMPUTE NennerF1=0.816 + 0.793 + 0.788 + 0.736 + 0.673 + 0.638 + 0.590.<br>EXECUTE.<br>COMPUTE ZählerF1=0.816 * f11_15 +0.793 * f11_12 +0.788 * f11_11 +0.736 * f11_9 +0.673 * f11_18 +0.638 * f11_13 +0.590 * f11_16.<br>EXECUTE.<br>COMPUTE GesamtscoreF1=ZählerF1/NennerF1.
Factor 2. Perceived usefulness of the Internet for health information searching	COMPUTE GesamtscoreF2=(0.864 * f11_2+0.855 * f11_1+0.760 * f11_5+0.720 * f11_14+0.700 * f11_4+0.600 * f11_3)/(0.864 +0.855 +0.760 +0.720 +0.700 +0.600).
Factor 3: Usefulness of the information gained from Internet health information searching	COMPUTE GesamtscoreF3=(0.789*f11_7+0.778*f11_8+0.767*f11_6)/(0.789+0.778+0.767).

Table 4

Factor name	Formula (SPSS-Syntax)
Factor 1: Health and nutrition awareness	COMPUTE NennerF42_F1=0.796+0.787+0.729+0.668.<br>EXECUTE.<br>COMPUTE ZählerF42_F1=0.796*F42_2+0.787*F42_3+0.729*F42_4+0.668*F42_1.<br>EXECUTE.<br>COMPUTE GesamtscoreF42_F1=ZählerF42_F1/NennerF42_F1.<br>EXECUTE.
Factor 2. Reluctance to make use of medical support	COMPUTE NennerF42_F2=0.829+0.774.<br>EXECUTE.<br>COMPUTE Zählerf42_F2=0.829*F42_5+0.774*F42_6.<br>EXECUTE.<br>COMPUTE GesamtscoreF42_F2=ZählerF42_F2/NennerF42_F2.<br>EXECUTE.

Table 5

Factor name	Formula (SPSS-Syntax)
Factor 1: Personal disposition of being-well-informed as a patient	COMPUTE ZählerF20=0.805*f20_8+0.788*f20_9+0.771*f20_3+0.737*f20_5+0.718*f20_4+0.700*f20_7+0.694*f20_1+0.689*f20_6+0.627*f20_2.<br>COMPUTE NennerF20=0.805+0.788+0.771+0.737+0.718+0.700+0.694+0.689+0.627.<br>EXECUTE.<br>COMPUTE GesamtscoreF20=ZählerF20/NennerF20.<br>EXECUTE.

Table 6

Factor name	Formula (SPSS-Syntax)
Factor 1: Situational influences on Internet health information searching	COMPUTE ZählerF12_Faktor1=0.912*f12_1+0.857*f12_2+0.613*f12_3.<br>EXECUTE.<br>COMPUTE NennerF12_Faktor1=0.912+0.857+0.613.<br>EXECUTE.<br>COMPUTE GesamtscoreF12_Faktor1=ZählerF12_Faktor1/NennerF12_Faktor1.<br>EXECUTE.
Factor 2. Normative influences on Internet health information searching	COMPUTE ZählerF12_Faktor2=0.956*f12_8+0.944*f12_7.<br>EXECUTE.<br>COMPUTE NennerF12_Faktor2=0.956+0.944.<br>EXECUTE.<br>COMPUTE GesamtscoreF12_Faktor2=ZählerF12_Faktor2/NennerF12_Faktor2.<br>EXECUTE.

Table F

Formula for calculating the effect sizes (Hedges' g) in Tables 2 – 7:

$$g = \frac{(\overline{X}_1 - \overline{X}_2)}{s_{pooled}}$$

$$s_{pooled} = \frac{\sqrt{(n_1 - 1)s_1^2 + (n_2 - 1)s_2^2}}{n_1 + n_2 - 2}$$

Abbreviations in the formula:

g	Hedges' g
s	standard deviation
s_1	standard deviation of group 1 (women) for calculation
s_2	standard deviation of group 2 (men) for calculation
n	sample size
n_1	sample size (valid n) of group 1 (women) for calculation
n_2	sample size (valid n) of group 2 (men) for calculation
$\overline{X}_1$	mean of group 1 (women) for calculation
$\overline{X}_2$	mean of group 2 (men) for calculation

Source (numeration according to the list of references):

74. Fröhlich M, Emrich E, Pieter A, Stark R. Outcome Effects and Effects Sizes in Sport Sciences. Int J Sports Science and Engineering 2009;03(03):175–179. [FREE full text] [WebCite Cache: 6XZfPCMel] [accessed 2015-04-06]
